# Aromatic Sulfonamides including a Sulfonic Acid Tail: New Membrane Impermeant Carbonic Anhydrase Inhibitors for Targeting Selectively the Cancer-Associated Isoforms

**DOI:** 10.3390/ijms23010461

**Published:** 2021-12-31

**Authors:** Simone Giovannuzzi, Mario D’Ambrosio, Cristina Luceri, Sameh Mohamed Osman, Marco Pallecchi, Gianluca Bartolucci, Alessio Nocentini, Claudiu T. Supuran

**Affiliations:** 1 NEUROFARBA Department, Section of Pharmaceutical and Nutraceutical Sciences, University of Florence, Polo Scientifico, Via U. Schiff 6, Sesto Fiorentino, 50019 Firenze, Italy; simone.giovannuzzi@unifi.it (S.G.); marco.pallecchi@unifi.it (M.P.); gianluca.bartolucci@unifi.it (G.B.); claudiu.supuran@unifi.it (C.T.S.); 2 NEUROFARBA Department, Section of Pharmacology and Toxicology, University of Florence, Viale Gaetano Pieraccini 6, 50100 Firenze, Italy; mario.dambrosio@unifi.it (M.D.); cristina.luceri@unifi.it (C.L.); 3 Chemistry Department, College of Science, King Saud University, P.O. Box 2455, Riyadh 11451, Saudi Arabia; smahmoud@ksu.edu.sa

**Keywords:** carbonic anhydrase, cancer, membrane-impermeability, inhibition

## Abstract

We report here a new drug design strategy for producing membrane-impermeant carbonic anhydrase (CA; EC 4.2.1.1) inhibitors selectively targeting the tumor-associated, membrane-bound human CAs IX and XII over off-target cytosolic isoforms. To date, this approach has only been pursued by including permanent positively charged pyridinium type or highly hydrophilic glycosidic moieties into the structure of aromatic sulfonamide CA inhibitors (CAIs). Aliphatic (propyl and butyl) sulfonic acid tails, deprotonated at physiological pH, were thus incorporated onto a benzenesulfonamide scaffold by a common 1,2,3-triazole linker and different types of spacers. Twenty such derivatives were synthesized and tested for their inhibition of target (hCAs IV, IX, and XII) and off-target CAs (hCAs I and II). Most sulfonate CAIs induced a potent inhibition of hCAs II, IX, and XII up to a low nanomolar K_I_ range (0.9–459.4 nM) with a limited target/off-target CA selectivity of action. According to the drug design schedule, a subset of representative derivatives was assessed for their cell membrane permeability using Caco-2 cells and a developed FIA-MS/MS method. The complete membrane impermeability of the sulfonate tailed CAIs (≥98%) validated these negatively charged moieties as being suitable for achieving, in vivo, the selective targeting of the tumor-associated CAs over off-target ones.

## 1. Introduction

The World Health Organization (WHO) has defined cancer (also named malignant tumors and neoplasms) as a group of diseases involving abnormal cell growth with the potential of invade other parts of the body and spread to other organs. Specific symptoms depend on the organ/tissue primarily affected by the tumor development, whilst systemic symptoms occur as a response of the body to the pathology and mainly include fatigue, skin changes, weight loss, and an inflammatory state [1]. Cancer is a leading cause of mortality worldwide, with about 9–10 million deaths per year [2]. The high mortality rate is chiefly attributable to failure in the treatment of metastatic tumor and the development of drug resistance. Solid tumor cells show an inadequate delivery of oxygen, that is, hypoxia, because of a spatial disorganization and flow-based disruption of abnormal vasculature [3,4]. In fact, in the tumor intensively proliferating and expanding, distance between cells and existing vasculatures increases, hampering oxygen diffusion and creating an even more hypoxic environment [5,6,7].

**Figure 1:**
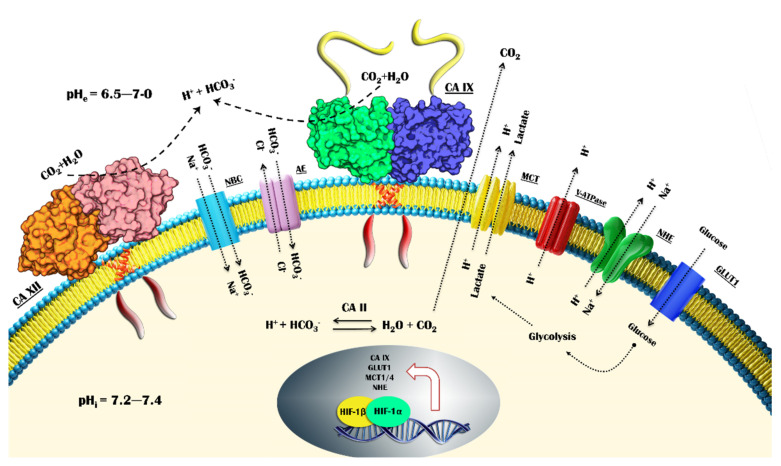
pH modulation system in hypoxic tumor cells. HIF-1 upregulates the expression of glucose transporters (GLUT1), glycolytic enzymes, and proteins involved in pH regulation: monocarboxylate transporter (MCT), V-type H+ATP (V-ATPase), Na+/H+ exchanger (NHE), bicarbonate co-transport (NBC), anion exchanger (AE), and carbonic anhydrase IX (CA IX) and XII (CA XII).

Hypoxia activates a variety of complex intracellular signaling pathways such as the major hypoxia-inducible factor (HIF) signaling cascade that fulfils the cell adaptive responses to hypoxia, triggering metabolic reprogramming, invasion, angiogenesis, immune suppression, resistance to apoptosis, and metastatization. The metabolic switch of cancer cells to glycolysis as a response to the inadequate oxygen supply increases the production of acid metabolites, including lactate, carbon dioxide, and protons [8,9]. To survive and reduce intracellular acidification, tumor cells activate complex molecular mechanisms involving ion exchangers, pumps, transporters, and carbonic anhydrases (CAs), which maintain a slightly alkaline intracellular pH (pHi) acidifying the extracellular environment (Figure 1) [10]. Pericellular accumulation of acidic metabolites results in the death of non-tumor cells and activates proteolytic enzymes that consume the extracellular matrix, promoting invasion and proliferation of cancer cells. Two particular CA isoforms, CAs IX and XII, are associated with cancer progression, metastasis, and impaired therapeutic responses. CAs IX and XII were in fact validated as alternative targets for chemotherapy of hypoxic solid tumors.

**Figure 2:**
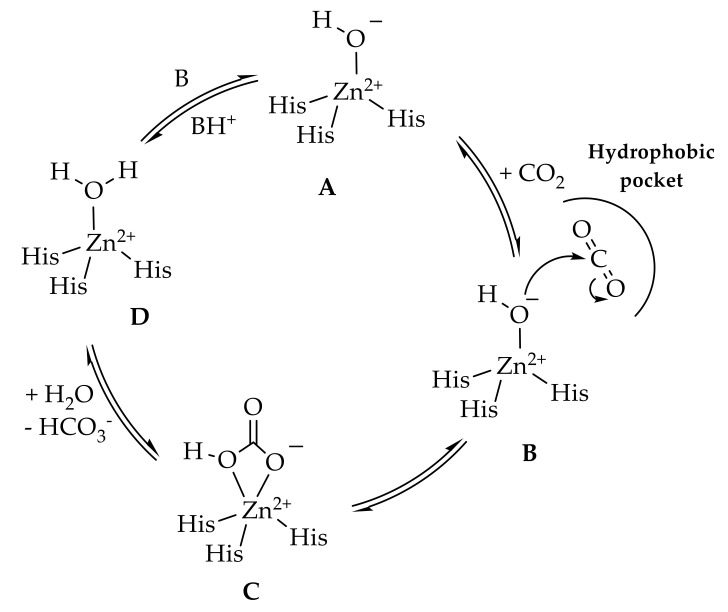
Schematic representation of the hCA catalytic mechanism.

The tumor-associated CAs are two of the 15 α-class isozymes identified in human (hCA), among which only 12 show catalytical activity. In particular, the catalysis of the reversible hydration of carbon dioxide (CO_2_) to produce bicarbonate and proton is the main physiological role of CAs, occuring by a two-step mechanism (Figure 2) (1): (i) the nucleophilic attack of the zinc-bound hydroxide ion (A) to a CO_2_ molecule present in the active site (B) to generate a HCO_3_^−^ ion (C), which is displaced by another H_2_O molecule (D); (ii) regeneration of catalytically active metal hydroxide species by a proton transfer reaction from the zinc-bound water to an exogenous proton acceptor or to an active site residue. The 15 hCA isoforms have different cellular localization, catalytic activity, and expression levels in normal vs. aberrantly functioning cells. Many of these isoforms are already validated targets for drug intervention to treat various disease and, yet, novel links to pathological disorders continue to be discovered [11,12,13,14].

Relevant for this study, CA IX and XII are involved in tumorigenesis, metastasis, and cancer progression [15]. CA IX was discovered by Pastorek et al. in 1994 [16], whilst CA XII was identified by Tureci et al. in 1998 [17]. A first unusual feature of CA IX and XII is their subcellular location: the two enzymes are transmembrane, multi-domain proteins incorporating a short intra-cytosolic tail, a transmembrane short domain, and an extracellular catalytic domain [18]. Furthermore, CA IX has an additional proteoglycan (PG) domain at its N-terminus. On the whole, this multi-domain structure makes CA IX a crucial protein in tumorigenesis and related processes in hypoxic tumors [19,20,21]. Indeed, CA IX and XII, overexpressed in hypoxic tumors upon HIF-α activation, contribute to acidifying extracellular pH, promoting cancer cell growth and metastasis. Moreover, recent proteomic studies suggests that CA IX shows a nuclear localization at an early stage, interacting with proteins involved in nuclear/cytoplasmic transport processes, gene transcription, and protein stability [22]. The precise role of these interactions is poorly understood to date, but may lead soon to significant developments for drug design strategies. Recently, suppression of CAIX activity was also shown to increase cellular reactive oxygen species accumulation and thus susceptibility to alterations in iron homeostasis, enhancing ferroptosis and significantly inhibiting tumor growth [23]. In contrast, a limited number of reports exist on the role of CA XII in tumors [24,25]. In 2019, Guerrini et al. showed that this isoform is regulated by the Hedgehog (Hh) pathway in several types of cancer [26,27]. Physiologically, the Hedgehog pathway controls organ development during embryogenesis, whereas in adults remains quiescent, except for tissue repairing [28,29]. Of interest, aberrations of this pathway occur in tumors, being responsible for tumorigenesis and cancer maintenance [30,31]. In 2020, Giuntini et al. demonstrated that CA XII inhibitors in hypoxia are able to lead to a reduction of both cells migration and invasion in melanoma cell lines [32]. In summary, the involvement of CA IX and XII in many pivotal processes of tumor development have made these enzymes sought and interesting targets for the development of new chemotherapeutics.

In the present study, we report the design and synthesis of a new series of benzenesulfonamide derivatives as potent CAIs capable of selectively targeting the cancer-associated CAs on the basis of their physico-chemical properties.

## 2. Results and Discussion

### 2.1. Drug Design and Chemistry

**Figure 3:**
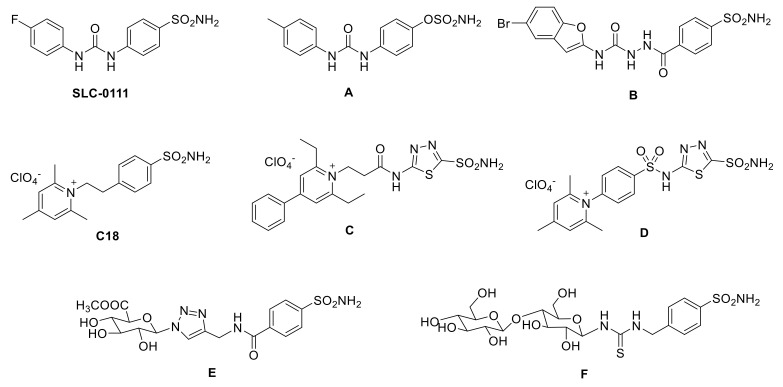
Structure of **SLC-0111** and its analogue derivatives, **A** and **B**; the membrane impermeant pyridinium CAIs, **C18**, **C**, and **D**; sugar-tailed CAIs, **E** and **F**.

A primary sulfonamide is the most effective and adopted zinc binding group (ZBG) for designing CA inhibitors [11,33]. A main issue of the first/second generation of sulfonamide CAIs, such as acetazolamide, dorzolamide, and brinzolamide, is the absence of isoform selectivity that impairs their systemic use for the treatment of the many pathologies in which CA isoforms are implicated. Although many other chemotypes, such as prodrug CAIs of the coumarin, and sulfocoumarin types have been discovered that have shown good efficacy and significant isoform selectivity, sulfonamides remain unbeaten in terms of inhibitory potency. As a result, many drug design strategies, most of which aimed to produce antitumor derivatives, have been developed with the aim of increasing the specificity of action of sulfonamide-like CAIs. In this context, the most efficient and adopted strategy is the tail approach, which is based on the mutations in the middle–outer rim of the active sites among the various CA isoforms and on the inclusion of specific groups in the aromatic sulfonamide CAI structure to selectively improve the ligand/target interactions. Among the great number of sulfonamides reported according to this strategy, only few compounds have been investigated in animal models, and only one derivate, **SLC-0111** (Figure 3), progressed to clinical trials, currently facing phase Ib/II for the treatment of advanced hypoxic tumors [34,35,36,37,38,39]. **SLC-0111** is an ureido-substituted benzenesulfonamide derivate that shows significant hCA IX and XII inhibitory properties in vitro (hCA IX, K_I_ = 45 nM; hCA XII, K_I_ = 4.5 nM), being much less effective as inhibitor of hCA I and II [39]. Being the first in class, **SLC-0111** has been used as a lead molecule for designing other compounds with a similar scaffold (e.g., **A** and **B** in Figure 3) [40,41,42,43,44,45,46,47,48,49].

**Figure 4:**
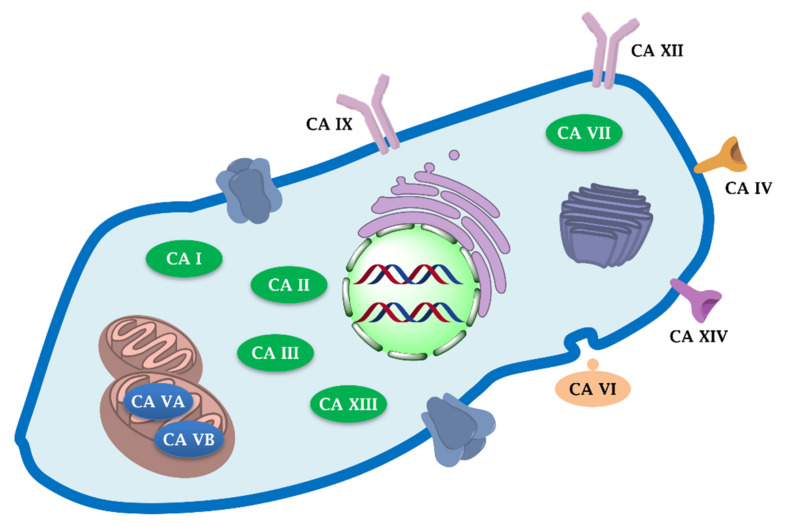
Subcellular localization of hCAs.

Prior to the development of **SLC-0111**, another drug design strategy was proposed, which is also based on the inclusion of specific molecular tails into the aromatic sulfonamide CAI structure. In this case, the tail is not included for specifically increasing the interactions with certain CAs but for providing the derivatives with physico-chemical properties that impair the cell membrane crossing. In fact, of the 12 catalytically active isoforms, 5 are cytosolic isozymes (CA I, II, III, VII, and XIII); 2 are present in mitochondria (CA VA and VB); 1 is secreted (CA VI); and 4 are membrane-associated, exposing the active site outwards (CA IV, IX, XII, and XIV) (Figure 4) [10,11].

As a result, CAIs bearing highly hydrophilic groups are membrane-impermeant and cannot interact with intracellular isozymes, promoting the selective targeting of membrane-associated CAs, among which CA IX and XII are overexpressed in the target hypoxic tumor context. Relevantly for this study, recent findings also pointed out a tumor-associated potential of CA IV (particularly in brain tumors). In fact, it was shown that CA IV mRNA expression is elevated in gliomas, renal cell carcinomas, thyroid cancers, and melanomas.

Up to now, this strategy was only pursued by incorporating permanent positive charges of the pyridinium type into the structure of aromatic sulfonamides (**C18**, **C**, and **D** in Figure 3) [50]. Compound **C18** represents the prototype of this type of CAI, demonstrated to possess a high selectivity for the membrane-associated (CA IV, IX, XII, and XIV) over cytosolic or mitochondrial CA isoforms [50]. In fact, due to their salt-like character, this type of inhibitor was unable to penetrate through biological membranes, as shown by ex vivo and in vivo perfusion experiments in rats. Another similar drug design approach included instead glycosidic moieties onto the structure of aromatic sulfonamide derivatives (e.g., **E** and **F** in Figure 3) that also produced membrane impermeability to be exploited for the selective targeting of membrane-associated CAs [51].

**Figure 5:**
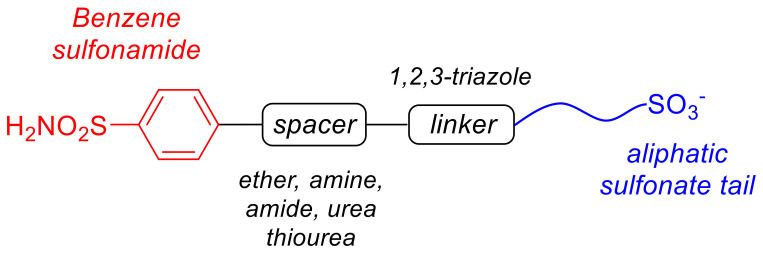
Design of benzenesulfonamides bearing aliphatic sulfonic acid tails.

To the best of our knowledge, there has been no investigation on a permanent negative charge as an approach for impairing membrane permeability of CAIs. Driven by the outcomes of virtual screening procedures applied to the identification of new potent CAIs, we selected aliphatic sulfonate moieties as tails to be included for the first time in benzenesulfonamide derivatives (Figure 5). The aliphatic sulfonate group as a tail was shown to enhance ligand/target interactions at the outer rim of the active site, where most charged amino acids are located. Moreover, the sulfonate, as a deprotonated sulfonic acid form, is permanently charged and suitable for increasing the CAI hydrophilicity up to hinder membrane penetration.

On the other hand, the high hydrophilicity and character of sulfonate groups shown to be deleterious for most chemical pathways attempted to include the permanently charged moiety in the CAI structure. As a result, a very versatile synthetic approach, such as the azide-alkyne Huisgen cycloaddition—Click Chemistry, was adopted as the only functioning chemical reaction to link the two portions of the molecule, namely, a benzenesulfonamide and the aliphatic sulfonic acid pendants. 1,2,3-Triazoles are amide bioisosters, stable and widely adopted linkers in the CAI research field that have been shown to positively impact the binding efficacy to several CA isoforms [52,53,54,55,56,57].

**Scheme 1:**
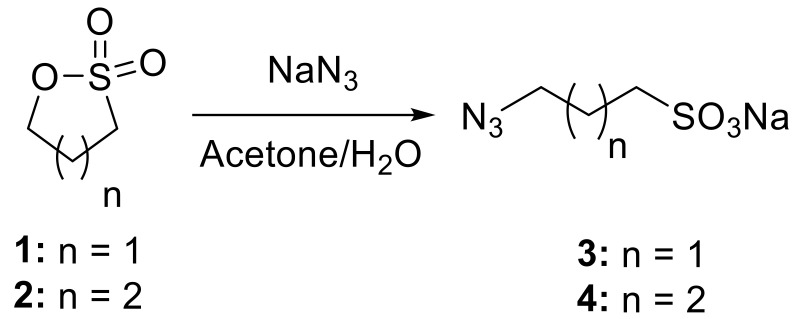
Synthesis of azide intermediates **3** and **4**.

Aliphatic sulfonate tails including an azide moiety were synthesized from 3-propanesultone **1** or 4-butanesultone **2** by reaction with sodium azide in H_2_O/acetone to produce the sodium sulfonates **3** and **4** with high yields and purity (Scheme 1).

**Scheme 2:**
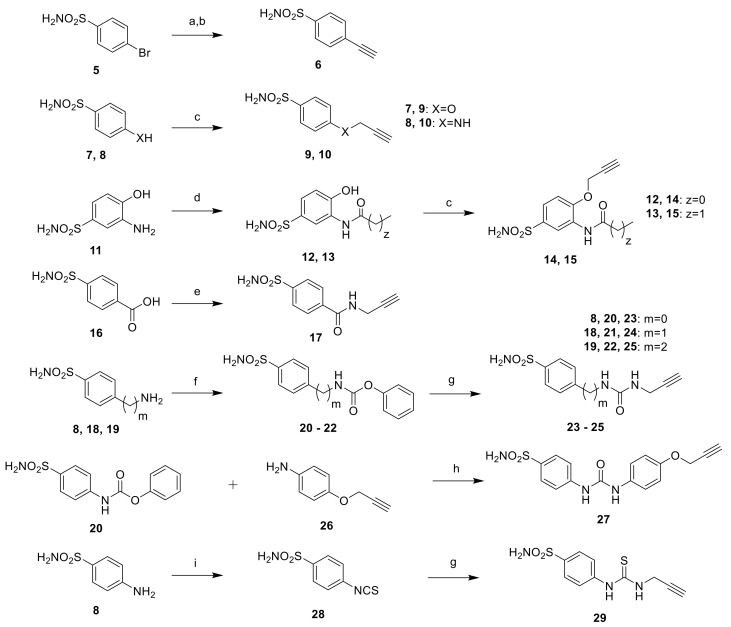
Synthetic pathways adopted to yield benzenesulfonamides bearing a terminal alkyne moiety **6**, **9**, **10**, **14**, **15**, **17**, **23**–**25**, **27**, and **29**. Reagents and conditions: (a) PdCl_2_[P(Ph)_3_]_2_, CuI, TMSA, Et_3_N, dry dioxane, 80 °C, o.n.; (b) K_2_CO_3_, MeOH, rt, o.n.; (c) propargyl bromide, K2CO3 (or dry pyridine), dry DMF, rt, 24–48 h; (d) acetic anhydride or propionic anhydride, EtOH, rt, 30 min; (e) propargyl amine, EDCI, DIPEA, dry DMF, 0 °C to rt, o.n.; (f) phenylchloroformate, K_2_CO_3_, acetone, 0 °C to rt, 3 h; (g) propargyl amine, DIPEA (cat), dry ACN, rt or reflux, 2 h; (h) DIPEA (cat), dry ACN, reflux; (i) thiophosgene, HCl 1 M, 0 °C to rt, 3 h.

To build the terminal alkyne counterparts for the Click Chemistry step, we planned different synthetic strategies to vary the spacer between the benzesulfonamide, chosen to provide a potent CA inhibitory action, and the 1,2,3-triazole, for working out extended structure–activity relationships (Scheme 2). 

**Scheme 3:**
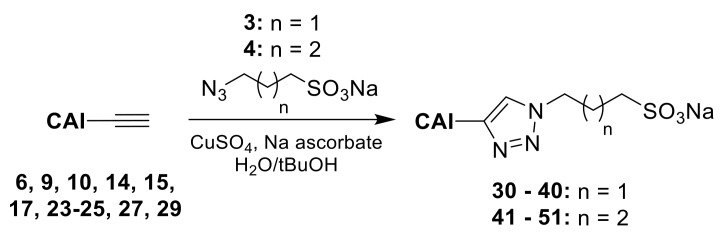
Huisgen azide-alkyne 1,3-dipolar cycloaddition to afford 1,2,3-triazoles **30**–**51**.

Specifically, compound **6** was obtained from 4-bromobenzenesulfonamide **5** by a Sonogashira reaction with CuCl_2_[P(Ph)_3_]_2_ as a catalyst. Nucleophilic substitution between intermediates **7** and **8** and propargyl bromide generated the corresponding ether **9** and amine **10**. The amine moiety of 4-sulfamoyl-2-aminophenol **11** was protected in the presence of acetic anhydride or propionic anhydride to produce amides **12** and **13**. The latter were reacted with propargyl bromide as well upon a nucleophilic substitution to obtain alkynes **14** and **15**. A coupling reaction between 4-sulfamoylbenzoic acid and propargyl amine produced amide **17**, using EDCI as a coupling reagent. Amines **8**, **18**, and **19** were activated to phenylcarbamates **20**–**22** with phenylchloroformate to successively produce the corresponding ureas **23**–**25** by reaction with propargyl amine. Carbamate **20** was also reacted with the freshly synthesized intermediate **26** to produce compound **27**. Isothiocyanate **28** was obtained from sulfanilamide **8** by reaction with thiophosgene and thus converted into thiourea **29** in the presence of propargyl amine. Finally, aliphatic azides **3** and **4** were reacted with terminal alkynes **6**, **9**, **10**, **14**, **15**, **17**, **23**–**25**, **27**, and **29** by a Huisgen azide-alkyne 1,3-dipolar cycloaddition (Click Chemistry) performed in H_2_O/tBuOH in the presence of CuSO_4_ and sodium ascorbate for the generation of the Cu(I) catalyst to produce 1,2,3-triazole **30**–**51** in high yield and purity (Scheme 3).

### 2.2. Carbonic Anhydrase Inhibiton

Derivatives **30**–**51** were tested for their inhibitory action against the target CA isoforms IX and XII, the potential antitumor target CA IV, and the off-target CAs I and II. Acetazolamide (AAZ) was used as standard CAI. CA I is the main off-target isoform for most therapeutic applications of CAIs, whilst CA II is considered off-target in tumor treatment to reduce side effects resulting from systematic CA inhibition as much as possible. The following structure–activity relationship (SAR) can be gathered from the inhibition data reported in Table 1.

The citosolyc CA I was inhibited by benzenesulfonamide derivatives **30**–**51** with inhibition constants (K_I_s) ranging in a medium nanomolar to low micromolar range (K_I_s 51.6–>10,000 nM). Interestingly, the ureido derivatives stood out as the most efficient CA I inhibitors (K_I_s 51.6–650.9 nM), among which a greater scaffold complication (**38**) produced the lowest K_I_ within the series (K_I_ of 51.6 nM). No relevant difference was detected between the propyl and butylsulfonate series. K_I_ values below 100 nM were also measured for both ether derivatives **31** (K_I_ of 82.7 nM) and **42** (K_I_ of 96.8 nM), and the propylsulfonate **30**, bearing a direct linkage between the triazole and the benzenesulfonamide moieties (K_I_ of 83.5 nM). Interestingly, *m*-substitutions of the benzenesulfonamide scaffold with acetammido and propionammido groups as in **39**, **40**, **50**, and **51** worsened the compounds CA I inhibitory action (K_I_s 3650.5–>10,000 nM), likely due to a further steric hindrance in the inner portion of the binding pocket that is deleterious within the narrower active site of CA I compared to other isoforms. 

The citosolyc hCA II was effectively inhibited by most triazole sulfonate derivatives with K_I_ values spanning in subnanomolar to medium nanomolar range (K_I_s 0.9–363.8 nM). Propylsulfonate derivatives generally showed a more efficient CA II inhibition than butylsulfonate compounds (K_I_ range 0.9–138.6 nM vs. 5.8–363.8 nM), except for the compound pair **37** and **48**, in which a longer tail produced a lower K_I_ value (5.8 nM vs. 21.4 nM). Interestingly, in the propylsulfonate subset, the lenghtening of the spacer by additional 1 or 2 carbon atoms, as in **35** and **36**, induced a slight inhibition reduction up to K_I_ values of 138.6 and 43.1 nM, with the *m*-acetammido **29** being the unique exception to this rule (K_I_ of 63.4 nM). Moreover, in the butylsulfonate subset, an acetammido substituent in *meta* position of the benzenesulfonamide (**50**) induced a reduction of CA II inhibition (K_I_ of 263.3 nM) when compared to the propionammido derivative **51** (K_I_ of 39.9 nM).

The membrane-bound CA IV is the less inhibited isoform in the kinetic study, probably due to its peculiar active site architecture and amino acid composition in comparison to most other hCA isoforms [57]. Again, butylsulfonate derivatives generally showed a less efficient CA IV inhibition than propylsulfonate compounds (K_I_ range 36.7–869.7 nM vs. 407.9–>10,000 nM). In particular, compounds **49**, **50**, and **51**, showing further steric hindrance both in the benzenesulfonamide scaffold and in the linker, stood out as the weakest CA IV inhibitors (K_I_s of 4540.8 nM, 3564.2 nM and >10,000 nM, respectively). Derivative **36**, bearing the longest spacer portion, resulted in being the least CAI of the propylsulfonate series in terms of inhibitory efficacy (K_I_ of 869.7 nM). In the latter series, very short (**31**, **32**) or very bulky (**38**) linker produced the best CA IV inhibition (K_I_s in the range 36.7–43.1 nM), while the absence of such a linker, as in **30**, impaired the inhibition up to a KI of 429.0 nM. 

The target CA IX resulted in being the second most efficiently inhibited isoform by sulfonate CAIs **30–51** with K_I_s in the range of 3.2–459.4 nM. As in CA I, no relevant K_I_ difference was detected between the propyl and butylsulfonate series, where potent inhibitors with up to single-digit nanomolar K_I_ values were identified. In detail, the first-in-class compound in terms of CA IX inhibition was **33**, which showed an amide linker and a propylsulfonate tail (K_I_ of 3.2 nM), whilst the absence of a spacer (**41**) or a thioureido linker (**48**) produced the best inhibitors of the butylsulfonate subset with K_I_s of 8.6 and 8.8 nM, respectively. Interestingly, a simple amine linker in the propylsulfonate series (**32**) was shown to be not tolerated with CA IX, inducing a K_I_ worsening up to 459.4 nM. *m*-Substitutions of the benzenesulfonamide did not significantly affect the compounds CA IX inhibitory efficacy, most probably as a result of the roomier active site of the cancer-associated isozymes in comparison to most other hCAs.

The trans-membrane CA XII was the most inhibited isoform by derivatives **30–51**, with KIs spanning in a flat range between 3.4 and 67.6 nM, with the amine-linked propylsulfonate derivative **32** as a unique exception (K_I_ of 433.1 nM). It can be speculated that the co-presence of a roomy active site with several Thr and Ser residues at the entrance of the binding cavity induces suitable and effective interactions between the amino acid alcoholic groups and the sulfonate moiety of the CAIs. Again, the length of the sulfonic tail was shown not to influence the CA inhibitory efficacy. It is interesting that an amine linker in the butylsulfonate subset produced the better CA XII inhibitor of the study (**43**, K_I_ of 3.4 nM). Only the *m*-propionammido compound **51** showed a single-digit nanomolar K_I_ of 7.3 nM within the butylsulfonate set. In fact, most other inhibitors showing a K_I_ below 10 nM belonged to the propylsulfonate group, in which compounds of almost all linker types reached a low nanomolar inhibitory efficacy, such as phenyltriazole **30**, amide **33**, urea **36**, and thiourea **37** (K_I_s of 6.7–9.5 nM).

As for selectivity of action against the target over off-target CAs (Table 2), all compounds exhibited remarkable I/IX and I/XII inhibitory specificity, as the calculated selectivity index (*SI*) spanned the ranges of 0.27–167 and 1.05–855, respectively. In detail, the best inhibitors with a propylsulfonate tail were the amide-linked **33** (*SI*: CA I/CA IX, 143; *SI*: CA I/CA XII, 48), the methylureido **35** (*SI*: CA I/CA IX, 11; *SI*: CA I/CA XII, 49), **39** (*SI*: CA I/CA IX, 41; *SI*: CA I/CA XII, 534), and **40** (*SI*: CA I/CA IX, 167; *SI*: CA I/CA XII, 194), having an amide substituent at the *m*-position of the aromatic ring. In the butylsulfonate subset, the amide-linked **43** (*SI*: CA I/CA IX, 36; SI: CA I/CA XII, 855) and the *m*-substituted **50** (*SI*: CA I/CA IX, 93; SI: CA I/CA XII, 189) and **51** (*SI*: CA I/CA IX, 15; SI: CA I/CA XII, 500) resulted as the most selective hCA IX and XII inhibitors over hCA I. In contrast, only four distinct sulfonamide derivatives produced a preferred inhibition of the target CA IX and XII over the main off-target hCA II. These inhibitors were **35** (*SI*: CA II/CA IX, 1.9; *SI*: CA II/CA XII, 8.4), **43** (*SI*: CA II/CA IX, 4.5; *SI*: CA II/CA XII, 107), **46** (*SI*: CA II/CA IX, 2.5; *SI*: CA II/CA XII, 10), and **50** (*SI*: CA II/CA IX, 3.2; *SI*: CA II/CA XII, 6.5). The amide-linked **43** from the butylsulfonate set showed a greatest selectivity for hCA IX and XII over the off target hCA I and II with better *SI* values than **AAZ** used as standard CAI.

### 2.3. Membrane Permeability Studies

The cell membrane permeability of CAIs bearing an aliphatic sulfonate group was evaluated using Caco-2 cells as an experimental model. The cells were exposed for 2 h at 37 °C to a final 50 μM concentration of six CAIs, namely, **33**, **34**, **38**, **39**, **41**, and **43**, selected as representatives of all types of included linker/spacer. A ligand concentration of 50 μM was used as averagely active in antiproliferative studies with cancer cell lines. No CAI affected cell viability after 2 h of exposure at a final concentration (viability >95%; data not shown). Thus, culture media and cell lysates were collected for MS/MS analyses. The permeation of each compound within Caco-2 cells was evaluated quantitatively by comparing the signal registered by a MS/MS method in the incubated samples (medium and lysate solutions) vs. the reference signal (culture medium at time 0: spiking solution). The polar characteristics of these compounds did not allow for the use of a classical liquid chromatography separation. Thus, a flow injection analysis (FIA) was carried out for each sample. The specificity in the compound determination was achieved by using MS/MS experiments, which allowed the monitoring of precursor-product ions characteristic transitions for each analyte (Table S1, Figures S1–S6, Supporting Information). In order to optimize the MS/MS condition and select the best product ions, we performed a series of energy resolved tandem mass spectrometry (ERMS) experiments. 

**Figure 6:**
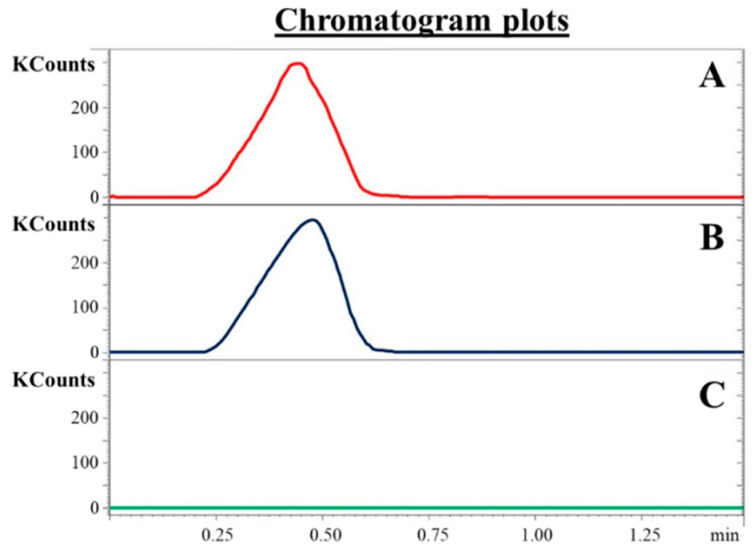
FIA-MS/MS profiles of (**A**) spiked solution (time 0), (**B**) medium solution, and (**C**) cell lysate solution after 2 h of incubation time with Caco-2 cells obtained from analysis of compound **38**. Ligand concentration of 50 μM in the spiked solution. Limit of detection: 2% of the initial concentration.

The MS/MS profiles of the spiked and medium solution of each compound showed the same signal abundance (≥98%, according to the detection limit), while no signal was detected in the cell lysate sample (≤2%, according to the limit of detection). The representative chromatogram plots of compound **38** are shown in Figure 6. These results demonstrated the effectiveness of the proposed drug design approach for achieving membrane impermeability.

## 3. Materials and Methods

### 3.1. Chemistry

Anhydrous solvents and all reagents were purchased from Merck, Fluorochem, and TCI. All reactions involving air- or moisture-sensitive compounds were performed under a nitrogen atmosphere using dried glassware and syringe techniques to transfer solutions. Nuclear magnetic resonance (^1^H-NMR, ^13^C-NMR) spectra were recorded using a Bruker Advance III 400 MHz spectrometer in DMSO-d_6_. Chemical shifts are reported in parts per million (ppm), and the coupling constants (J) are expressed in Hertz (Hz). Splitting patterns are designated as follows: s, singlet; d, doublet; t, triplet; q, quadruplet; m, multiplet; bs, broad singlet; dd, doublet of doublets. The assignment of exchangeable protons was confirmed by the addition of D_2_O. Analytical thin-layer chromatography (TLC) was carried out on Sigma-Aldrich silica gel F-254 plates. Flash chromatography purifications were performed on Sigma-Aldrich Silica gel 60 (230–400 mesh ASTM) as the stationary phase, and ethyl acetate/n-hexane or MeOH/DCM were used as eluents. Melting points (mp) were measured in open capillary tubes with a Gallenkamp MPD350.BM3.5 apparatus and were uncorrected. The solvents used in mass spectrometry analysis were acetone and acetonitrile (Chromasolv grade), purchased from Sigma-Aldrich (Milan, Italy), and mQ water 18 MΩ cm, obtained from Millipore’s Simplicity system (Milan, Italy). The HPLC–MS and MS/MS analysis was carried out using a Varian 500-MS ion trap system (Palo Alto, CA, USA) equipped by two Prostar 210 pumps, a Prostar 410 autosampler, and an electrospray source (ESI) operating in negative ions. Stock solutions of analytes were prepared in acetone at 1.0 mg mL^−1^ and stored at 4 °C. Working solutions of each analyte were freshly prepared by diluting stock solutions in a mixture of mQ water/acetonitrile 1:1 (*v*/*v*) up to a concentration of 1.0 µg mL^−1^. The mass spectra of each analyte were acquired by introducing, via syringe pump at 10 µL min^−1^, the working solution. Raw data were collected and processed by Varian Workstation Vers. 6.8 software.

#### 3.1.1. General Synthetic Procedure of Sodium 3-Azidopropane-1-sulfonate (**3**) and Sodium 4-Azidobutane-1-sulfonate (**4**)

A solution of NaN_3_ (0.95 g, 1 equiv) in H_2_O (4 mL) was added dropwise at RT to a stirring solution of sultone **1** or **2** (1 equiv) in acetone (20 mL). The resulting mixture was stirred for 4 h at rt. The solvent was removed under vacuum and the resulting sodium salt was suspended in Et_2_O and filtered to obtain the title compound as a white powder.

*Sodium 3-azidopropane-1-sulfonate (***3***)*

Yield 98%; m.p. 163–166 °C; silica gel TLC Rf 0.01 (MeOH/DCM 10% *v*/*v*); δ_H_ (400 MHz, DMSO-d_6_): 3.40 (t, *J* = 6.2 Hz, 2H, C*H*_2_), 2.45 (t, *J* = 6.9 Hz, 2H, C*H*_2_), 1.81 (m, 2H, C*H*_2_); *δ_C_ (100 MHz, DMSO-d_6_): 49.3, 48.0, 23.0*.

*Sodium 4-azidobutane-1-sulfonate (***4***)*

Yield 74%; m.p. 187–189 °C; silica gel TLC Rf 0.01 (MeOH/DCM 10% *v*/*v*); δ_H_ (400 MHz, DMSO-d_6_): 3.33 (t, *J* = 6.4 Hz, 2H, C*H*_2_), 2.44 (t, *J* = 6.7 Hz, 2H, C*H*_2_), 1.27 (m, 4H, 2 × C*H*_2_); δ_C_ (100 MHz, DMSO-d_6_): 51.9, 47.0, 24.1, 20.2.

#### 3.1.2. Synthesis of 4-Ethynylbenzenesulfonamide (**6**)

Trimethylsilylacetylene (1.87 g, 2 equiv), PdCl_2_(P(Ph)_3_)_2_ (0.44 g, 0.05 equiv), and CuI (0.24 g, 0.1 equiv) were added to a solution of 4-bromobenzenesulfonamide **5** (3 g, 1 equiv) in dry dioxane (30 mL) under nitrogen atmosphere, and the suspension was heated o.n. at 80 °C. The solvent was partially removed under vacuum, and the reaction mixture was quenched with slush and extracted in EtOAc (3 × 30 mL). The collected organic phase was dried over anhydrous Na_2_SO_4_, filtered, and evaporated to produce a brown oil residue. K_2_CO_3_ (5.8 g, 3 equiv) was added to a solution of the residue in MeOH (20 mL), and the suspension was stirred o.n. at RT. The solvent was then removed under vacuum and the resulting crude product **6** was purified by silica gel chromatography (EtOAc/hexane 20% to 50%) to produce **6** as a powder. Yield 40%; m.p. 175–176 °C; silica gel TLC Rf 0.51 (EtOAc/n-Hexane 50% *v*/*v*); δ_H_ (400 MHz, DMSO-d_6_): 7.84 (d, *J* = 8.5 Hz. 2H Ar-*H*), 7.70 (d, *J* = 8.5 Hz, 2H, Ar-*H*), 7.50 (s, 2H, exchange with D_2_O, SO_2_N*H*_2_), 4.49 (s, 1H, C*H*); δ_C_ (125 MHz, CDCl_3_-d_1_): 109.1, 126.0, 129.1, 131.3, 138.2, 143.5.

#### 3.1.3. Synthesis of 4-(Prop-2-yn-1-yloxy)benzenesulfonamide (**9**)

Propargyl bromide 80% (1.2 equiv) was added dropwise to a suspension of 4-hydroxybenzenesulfonamide **7** (1.5 g, 1 equiv) and K_2_CO_3_ (1.2 equiv) in dry DMF (3 mL) under nitrogen atmosphere; then, the mixture was stirred o.n. at RT. The suspension was quenched with slush and HCl 6M, and the resulting precipitate was filtered to obtain product **9** as white powder. Yield 97%; m.p. 248–250 °C; silica gel TLC Rf 0.46 (MeOH/DCM 5% *v*/*v*); δ_H_ (400 MHz, DMSO-d_6_): 7.79 (d, *J* = 8.4 Hz, 2H, Ar-*H*), 7.25 (s, 2H, exchange with D_2_O, SO_2_N*H*_2_), 7.16 (d, *J* = 8.4 Hz, 2H, Ar-*H*), 4.92 (s, 2H, C*H*_2_), 3.64 (s, 1H, C*H*); δ_C_ (100 MHz, DMSO-d_6_): 161.0, 136.0, 126.1, 114.6, 78.7, 76.4, 56.9.

#### 3.1.4. Synthesis of 4-(Prop-2-yn-1-ylamino)benzenesulfonamide (**10**)

Dry pyridine (1.5 equiv) and propargyl bromide 80% (1.5 equiv) were added dropwise to a stirred solution of sulfanilamide **8** (2 g, 1 equiv) in dry DMF (7 mL) under nitrogen atmosphere, and the reaction mixture was stirred at RT for 48 h. The reaction mixture was quenched with slush and extracted in EtOAc (3 × 25 mL). The combined organic layers were washed with brine, dried over anhydrous Na_2_SO_4_, filtered, and evaporated to obtain a brown oil residue that was purified by silica gel chromatography (EtOAc/n-Hexane, 20% to 50%) to give **10** as a white powder. Yield 32%; m.p. 218–221 °C; silica gel TLC Rf 0.28 (EtOAc/n-Hexane 50% *v/v*); δ_H_ (400 MHz, DMSO-d_6_): 7.57 (d, *J* = 8.6 Hz, 2H, Ar-*H*), 6.99 (s, 2H, exchange with D_2_O, SO_2_N*H*_2_), 6.75 (t, *J* = 5.1 Hz, 1H, exchange with D_2_O, N*H*), 6.71 (d, *J* = 8.6 Hz, 2H, Ar-*H*), 3.96 (dd, *J* = 5.1 2.3 Hz, 2H, C*H*_2_), 3.13 (t, *J* = 2.3 Hz, 1H, C*H*); δ_C_ (100 MHz, DMSO-d_6_): 150.8, 129.3, 127.3, 112.5, 79.5, 73.2, 29.8.

#### 3.1.5. General Synthesis of N-(2-Hydroxy-5-sulfamoylphenyl)acetamide (**12**) and N-(2-Hydroxy-5-sulfamoylphenyl)propionamide (**13**)

The appropriate anhydride (1.2 equiv) was added dropwise to a suspension of 3-amino-4-hydroxybenzenesulfonamide **11** (1.0 g, 1 equiv) in EtOH (15 mL). The reaction mixture was stirred at RT for 30 min. The solvent was removed under vacuum, and the residue was treated with slush and HCl 6M. The resulting suspension was filtered and washed with Et_2_O to obtain **12** and **13** as powders.

*N-(2-Hydroxy-5-sulfamoylphenyl)acetamide (***12***)*

Yield 91%; m.p. 190–192 °C; silica gel TLC Rf 0.33 (MeOH/DCM 10% *v/v*); δ_H_ (400 MHz, DMSO-d_6_): 10.74 (s, 1H, exchange with D_2_O, O*H*), 9.37 (s, 1H, exchange with D_2_O, CON*H*), 8.39 (s, 1H, Ar-*H*), 7.42 (d, *J* = 8.5 Hz, 1H, Ar-*H*), 7.16 (s, 2H, exchange with D_2_O, SO_2_N*H*_2_), 6.98 (d, *J* = 8.5 Hz, 1H, Ar-*H*), 2.14 (s, 3H C*H*_3_); δ_C_ (100 MHz, DMSO-d_6_): 169.3, 151.6, 132.5, 128.6, 121.8, 119.2, 115.3, 25.1.

*N-(2-Hydroxy-5-sulfamoylphenyl)propionamide (***13***)*

Yield 74%; m.p. 201–203°C; silica gel TLC Rf 0.19 (MeOH/DCM 10% *v*/*v*); δ_H_ (400 MHz, DMSO-d_6_): 10.67 (s, 1H, exchange with D_2_O, O*H*), 9.29 (s, 1H, exchange with D_2_O, CON*H*), 8.43 (s, 1H, Ar-*H*), 7.43 (d, *J* = 7.3 Hz, 1H, Ar-*H*), 7.17 (s, 2H, exchange with D_2_O, SO_2_N*H*_2_), 7.00 (d, *J* = 7.3 Hz, 1H, Ar-*H*), 2.46 (q, *J* = 7.0 Hz, 2H C*H*_2_), 1.11 (t, *J* = 7.0 Hz, 3H, C*H*_3_); δ_C_ (100 MHz, DMSO-d_6_): 172.7, 151.1, 132.2, 128.0, 122.1, 118.6, 115.9, 29.4, 11.3.

#### 3.1.6. General Synthesis for N-(2-(Prop-2-yn-1-yloxy)-5-sulfamoylphenyl)acetamide (**14**) and N-(2-(Prop-2-yn-1-yloxy)-5-sulfamoylphenyl)propionamide (**15**)

Propargyl bromide 80% (1.2 equiv) was added dropwise to a suspension of K_2_CO_3_ (1.2 equiv) and compound **12** or **13** (0.5 g, 1 equiv) in dry DMF (2 mL) under nitrogen atmosphere. The reaction mixture was stirred o.n. at RT and then quenched with slush and HCl 6M. The resulting precipitate was filtered to obtain products **14** and **15** as white powders.

*N-(2-(Prop-2-yn-1-yloxy)-5-sulfamoylphenyl)acetamide (***14***)*

Yield 90%; m.p. 243–245 °C; silica gel TLC Rf 0.51 (MeOH/DCM 10% *v*/*v*); δ_H_ (400 MHz, DMSO-d_6_): 9.41 (s, 1H, exchange with D_2_O, CON*H*), 8.52 (s, 1H, Ar-*H*), 7.56 (d, *J* = 8.0 Hz, 1H, Ar-*H*), 7.29 (d, *J* = 8.0 Hz, 1H, Ar-*H*), 7.28 (s, 2H, exchange with D_2_O, SO_2_N*H*_2_), 5.00 (s, 2H, C*H*_2_), 3.70 (s, 1H, C*H*), 2.14 (s, 3H C*H*_3_); δ_C_ (100 MHz, DMSO-d_6_): 168.9, 155.9, 132.2, 127.1, 121.7, 115.2, 114.8, 78.7, 76.4, 56.9, 24.0.

*N-(2-(Prop-2-yn-1-yloxy)-5-sulfamoylphenyl)propionamide (***15***)*

Yield 81%; m.p. 260–262 °C; silica gel TLC Rf 0.12 (MeOH/DCM 5% *v*/*v*); δ_H_ (400 MHz, DMSO-d_6_): 9.30 (s, 1H, exchange with D_2_O, CON*H*), 8.54 (s, 1H, Ar-*H*), 7.58 (d, *J* = 8.4 Hz, 1H, Ar-*H*), 7.29 (d, *J* = 7.3 Hz, 1H, Ar-*H*), 7.28 (s, 2H, exchange with D_2_O, SO_2_N*H*_2_), 5.00 (s, 2H, C*H*_2_), 3.69 (s, 1H, C*H*), 2.46 (q, *J* = 7.3 Hz, 2H C*H*_2_), 1.11 (t, *J* = 7.3 Hz, 3H, C*H*_3_); δ_C_ (100 MHz, DMSO-d_6_): 172.4, 155.9, 132.2, 127.1, 121.7, 115.2, 114.8, 78.7, 76.4, 56.9, 30.6, 10.1.

#### 3.1.7. Synthesis of N-(Prop-2-yn-1-yl)-4-sulfamoylbenzamide (**17**)

EDCI (2 equiv) and propargyl amine (1.1 equiv) were added to a solution of 4-sulfamoylbenzoic acid **16** (1 g, 1 equiv) in dry DMF (3 mL) at RT under nitrogen atmosphere. DIPEA (3 equiv) was thus added dropwise to the mixture. The resulting solution was stirred o.n. at RT. The reaction mixture was quenched with slush and HCl 6M to up to pH 2, and the resulting precipitate was filtered and washed with Et_2_O to afford **17** as a white powder. Yield 69%; m.p. 266–268 °C; silica gel TLC Rf; δ_H_ (400 MHz, DMSO-d_6_): 9.16 (t, *J* = 4.9 Hz, 1H, exchange with D_2_O, CON*H*), 8.03 (d, *J* = 8.4 Hz, 2H Ar-*H*), 7.93 (d, *J* = 8.4 Hz, 2H, Ar-*H*), 7.52 (s, 2H, exchange with D_2_O, SO_2_N*H*_2_), 4.10 (d, *J* = 3.5 Hz, 2H, C*H*_2_), 3.18 (t, *J* = 3.5 Hz, 1H, C*H*); δ_C_ (100 MHz, DMSO-d_6_): 165.62, 147.14, 137.34, 128.64, 126.37, 81.67, 73.81, 29.03.

#### 3.1.8. General Synthesis for Phenyl (4-Sulfamoylphenyl)carbamate (**20**), Phenyl (4-Sulfamoylbenzyl)carbamate (**21**) and Phenyl (4-Sulfamoylphenetyl)carbamate (**22**)

Phenylchloroformate (1.5 equiv) was added dropwise at 0 °C to a suspension of amine **8**, **18**, or **19** (0.5 g, 1 equiv) and K_2_CO_3_ (1.5 equiv) in acetone (30 mL), and the reaction mixture was stirred for 3 h at RT. The solvent was removed under vacuum, and the residue treated with slush and HCl 6M. The suspension was filtered to obtain **20**–**22** as white powders.

*Phenyl (4-sulfamoylphenyl)carbamate (***20***)*

Yield 95%; m.p. 281–283 °C; silica gel TLC Rf 0.38 (MeOH/DCM 6% *v*/*v*); δ_H_ (400 MHz, DMSO-d_6_): 10.66 (s, 1H, exchange with D_2_O, CON*H*), 7.80 (d, *J* = 8.3 Hz, 2H, Ar-*H*), 7.68 (d, *J* = 8.3 Hz, 2H, Ar-*H*), 7.47 (t, *J* = 8 Hz, 2H Ar-*H*), 7.29 (m, 5H, exchange with D_2_O, SO_2_N*H*_2_ + Ar-*H*); δ_C_ (100 MHz, DMSO-d_6_): 151.3, 150.0, 141.2, 136.5, 129.4, 129.1, 125.5, 121.6, 118.0.

*Phenyl (4-sulfamoylbenzyl)carbamate (***21***)*

Yield 87%; m.p. 256–257 °C; silica gel TLC Rf 0.48 (MeOH/DCM 10% *v*/*v*); δ_H_ (400 MHz, DMSO-d_6_): 8.42 (t, *J* = 5.5 Hz, 1H, exchange with D_2_O, CON*H*), 7.83 (d, *J* = 7.1 Hz, 2H, Ar-*H*), 7.52 (d, *J* = 7.1 Hz, 2H, Ar-*H*), 7.40 (m, 4H, Ar-*H*), 7.36 (d, 2H, exchange with D_2_O, SO_2_N*H*_2_), 7.23 (t, *J* = 7.4 Hz, 1H, Ar-*H*), 7.15 (d, *J* = 7.4 Hz, 2H, Ar-*H*), 4.37 (d, *J* = 5.5 Hz, 2H, C*H*_2_); δ_C_ (100 MHz, DMSO-d_6_): 155.0, 151.3, 141.7, 141.1, 129.1, 128.2, 127.2, 125.5, 121.6, 45.1.

*Phenyl (4-sulfamoylphenetyl)carbamate (***22***)*

Yield 94%; m.p. 235–238 °C; silica gel TLC Rf 0.62 (MeOH/DCM 10% *v*/*v*); δ_H_ (400 MHz, DMSO-d_6_): 7.88 (t, *J* = 5.6 Hz, 1H, exchange with D_2_O, CON*H*), 7.79 (d, *J* = 7.7 Hz, 2H, Ar-*H*), 7.46 (d, *J* = 7.7 Hz, 2H, Ar-*H*), 7.38 (d, *J* = 7.7 Hz, 2H, Ar-*H*), 7.33 (s, 2H, exchange with D_2_O, SO_2_N*H*_2_), 7.21 (t, *J* = 7.7 Hz, 1H, Ar-*H*), 7.08 (d, *J* = 7.7 Hz, 2H, Ar-*H*); δ_C_ (100 MHz, DMSO-d_6_): 153.4, 151.3, 142.6, 140.9, 129.1, 128.3, 128.0, 125.5, 121.6, 39.9, 35.0.

#### 3.1.9. General Synthesis for 4-(3-(Prop-2-yn-1-yl)ureido)benzenesulfonamide (**23**), 4-((3-(Prop-2-yn-1-yl)ureido)methyl)benzenesulfonamide (**24**) and 4-((3-(Prop-2-yn-1-yl)ureido)ethyl)benzenesulfonamide (**25**)

Propargyl amine (1.5 equiv) was added to a solution of the ureido derivatives **20–22** (1 equiv) and DIPEA (cat) in dry ACN (10 mL) under nitrogen atmosphere. The reaction mixture was stirred at reflux for 2h and then quenched with slush and HCl 1M. The precipitate was filtered and washed with Et_2_O to afford **23–25** as white powders.

*4-(3-(Prop-2-yn-1-yl)ureido)benzenesulfonamide (***23***)*

Yield 89%; m.p. 235–237 °C; silica gel TLC Rf 0.39 (EtOAc/n-Hexane 50% *v*/*v*); δ_H_ (400 MHz, DMSO-d_6_): 9.02 (s, 1H, exchange with D_2_O, N*H*CON*H*), 7.70 (d, *J* = 8.3 Hz, 2H, Ar-*H*), 7.57 (d, *J* = 8.3 Hz, 2H, Ar-*H*), 7.19 (s, 2H, exchange with D_2_O, SO_2_N*H*_2_), 6.66 (d, *J* = 5.3 Hz, 1H, exchange with D_2_O, N*H*CON*H*), 3.92 (d, *J* = 5.3 Hz, 2H, C*H*_2_), 3.15 (s, 1H, C*H*); δ_C_ (100 MHz, DMSO-d_6_): 154.3, 142.6, 136.5, 129.4, 118.0, 80.1, 73.2, 32.0.

*4-((3-(Prop-2-yn-1-yl)ureido)methyl)benzenesulfonamide (***24***)*

Yield 59%; m.p. 193–196 °C; silica gel TLC Rf 0.41 (EtOAc/n-Hexane 50% *v*/*v*); δ_H_ (400 MHz, DMSO-d_6_): 7.98 (d, *J* = 7.3 Hz, 2H, Ar-*H*), 7.64 (d, *J* = 7.3 Hz, 2H, Ar-*H*), 7.52 (s, 2H, exchange with D_2_O, SO_2_N*H*_2_), 6.84 (s, 1H, exchange with D_2_O, N*H*CON*H*), 6.61 (s, 1H, exchange with D_2_O, N*H*CON*H*), 4.50 (s, 2H, C*H*_2_), 4.03 (s, 2H, C*H*_2_), 3.29 (s, 1H, C*H*); δ_C_ (100 MHz, DMSO-d_6_): 157.6, 141.7, 141.2, 128.2, 127.2, 80.1, 73.2, 44.4, 32.0.

*4-((3-(Prop-2-yn-1-yl)ureido)ethyl)benzenesulfonamide (***25***)*

Yield 74%; m.p. 200–202 °C; silica gel TLC Rf 0.44 (EtOAc/n-Hexane 50% *v*/*v*); δ_H_ (400 MHz, DMSO-d_6_): 7.77 (d, *J* = 7.8 Hz, 2H, Ar-*H*), 7.41 (d, *J* = 7.8 Hz, 2H, Ar-*H*), 7.31 (s, 2H, exchange with D_2_O, SO_2_N*H*_2_), 6.24 (t, *J* = 5.7 Hz, 1H, exchange with D_2_O, N*H*CON*H*), 6.03 (t, *J* = 5.7 Hz, 1H, exchange with D_2_O, N*H*CON*H*), 3.79 (dd, J = 5.7 2.5 Hz, 2H, C*H*_2_), 3.28 (q, *J* = 6.9 5.7 Hz, 2H, C*H*_2_), 3.06 (t, *J* = 2.5 Hz, 1H, C*H*), 2.78 (t, *J* = 6.9 Hz, 2H, C*H*_2_); δ_C_ (100 MHz, DMSO-d_6_): 157.6, 142.6, 140.9, 128.3, 128.0, 80.1, 73.2, 41.5, 34.6, 32.0.

#### 3.1.10. Synthesis of 4-(Prop-2-yn-1-yloxy)aniline (**26**)

K_2_CO_3_ (1.2 equiv) and propargyl bromide 80% (1.5 equiv) were added to a solution of 4-acetamidophenol (0.9 g, 1 equiv) in dry DMF (2 mL). The reaction mixture was stirred at 60 °C for 6h under nitrogen atmosphere. The reaction mixture was thus quenched with slush and NaOH 5M, and the resulting precipitate was filtered to obtain a white powder in quantitative yield. HCl (5 equiv) was added to a suspension of the previously obtained powder in EtOH (10 mL), and the suspension was refluxed o.n. The reaction mixture was cooled and quenched with slush and NaOH up to pH 10. The precipitate was filtered to obtain compound **26** as a grey powder. Yield 66%; m.p. 262–265 °C; silica gel TLC Rf 0.52 (MeOH/DCM 10% *v*/*v*); δ_H_ (400 MHz, DMSO-d_6_): 6.72 (d, *J* = 7.9 Hz, 2H, Ar-*H*), 6.53 (d, *J* = 7.9 Hz, 2H, Ar-*H*), 4.68 (s, 2H, exchange with D_2_O, N*H*_2_), 4.61 (s, 2H, C*H*_2_), 3.49 (s, 1H, C*H*); δ_C_ (100 MHz, DMSO-d_6_): 147.8, 140.7, 115.8, 115.1, 78.7, 76.4, 56.9.

#### 3.1.11. Synthesis of 4-(3-(4-(Prop-2-yn-1-yloxy)phenyl)ureido)benzenesulfonamide (**27**)

Compound **26** (0.17 g, 1.1 equiv) was added to a solution of phenyl (4-sulfamoylphenyl)carbamate **20** (0.3 g, 1 equiv) and DIPEA (cat) in dry ACN (8 mL) under nitrogen atmosphere, and the reaction mixture was heated at reflux for 4 h. The reaction mixture thus was quenched with slush and HCl 1M, and the resulting precipitate was filtered and washed with Et_2_O to afford **27** as a grey powder. Yield 65%; m.p. 273–275 °C; silica gel TLC Rf 0.11 (MeOH/DCM 10% *v*/*v*); δ_H_ (400 MHz, DMSO-d_6_): 9.07 (s, 1H, exchange with D_2_O, N*H*CON*H*), 8.70 (s, 1H, exchange with D_2_O, N*H*CON*H*), 7.74 (d, *J* = 8.6 Hz, 2H, Ar-*H*), 7.62 (d, *J* = 8.6 HZ, 2H, Ar-*H*), 7.40 (d, *J* = 8.4 Hz, 2H, Ar-*H*), 7.24 (s, 2H, exchange with D_2_O, SO_2_N*H*_2_), 6.96 (d, *J* = 8.4 Hz, 2H, Ar-*H*), 4.77 (d, *J* = 1.9 Hz, 2H, C*H*_2_), 3.58 (t, J = 1.9 Hz, 1H, C*H*); δ_C_ (100 MHz, DMSO-d_6_): 153.4, 152.9, 142.6, 136.5, 131.7, 129.4, 119.8, 118.0, 114.5, 78.7, 76.5, 56.9.

#### 3.1.12. Synthesis of 4-Isothiocyanatebenzenesulfonamide (**28**)

Thiophosgene (1.25 equiv) was added dropwise at 0 °C to a solution of sulfanilamide (3 g, 1 equiv) in HCl 1M (20 mL), and the mixture was stirred at RT for 3h. After that, the suspension was quenched with slush and HCl 1M, and the resulting precipitate was filtered and washed with H_2_O to give **28** in high yield and purity as a yellow powder. Yield 94%; m.p. 212–214 °C; silica gel TLC Rf 0.83 (MeOH/DCM 10% *v*/*v*); δ_H_ (400 MHz, DMSO-d_6_): 7.88 (d, *J* = 8.3 Hz, 2H, Ar-*H*), 7.63 (d, J = 8.3 Hz, 2H, Ar-*H*), 7.52 (s, 2H, exchange with D_2_O, SO_2_N*H*_2_); δ_C_ (100 MHz, DMSO-d_6_): 142.7, 136.9, 134.7, 128.6, 124.6.

#### 3.1.13. Synthesis of 4-(3-(Prop-2-yn-1-yl)thioureido)benzenesulfonamide (**29**)

Propargylamine (1.2 equiv) was added to a solution of isothiocyanate (1 g, 1 equiv) in dry ACN (8 mL) under nitrogen atmosphere, and the resulting solution was stirred for 2 h at RT. Slush and HCl 1M were added to the mixture and the resulting precipitate was filtered and washed with Et_2_O to produce **29** as a white powder. Yield 90%; m.p. 235–238 °C; silica gel TLC Rf 0.42 (MeOH/DCM 10% *v*/*v*); δ_H_ (400 MHz, DMSO-d_6_): 9.98 (s, 1H, exchange with D_2_O, N*H*CSN*H*), 8.32 (s, 1H, exchange with D_2_O, N*H*CSN*H*), 7.77 (d, *J* = 8.1 Hz, 2H, Ar-*H*), 7.68 (d, *J* = 8.1 Hz, 2H, Ar-*H*), 7.31 (s, 2H, exchange with D_2_O, SO_2_N*H*_2_), 4.34 (s, 2H, C*H*_2_), 3.22 (s, 1H, C*H*); δ_C_ (100 MHz, DMSO-d_6_): 179.5, 141.7, 136.9, 129.5, 122.9, 80.1, 73.2, 37.1.

#### 3.1.14. General Synthesis of Triazole Derivatives **30**–**51**

Alkyne derivatives **6**, **9**–**10**, **14**–**15**, **17**, **23**–**25**, **27,** and **29** (1 equiv) were added to a suspension of **3** or **4** (0.2 g, 1 equiv) in tBuOH (3 mL). A suspension of CuSO_4_ ⋅ 5H_2_O (0.1 equiv) and sodium ascorbate (0.5 equiv) in water (3 mL) was added to the reaction mixture, which was stirred overnight at RT, and therefore treated with an additional amount of water (10 mL). The resulting suspension was filtered through celite, and thus concentrated under vacuum. The residue was triturated with acetone to produce the crude **30**–**51**, which were purified by silica gel chromatography (MeOH/DCM, 5% to 20%) to produce the pure powders by good yields.

*Sodium 3-(4-(4-sulfamoylphenyl)-1H-1,2,3-triazol-1-yl)propane-1-sulfonate (***30***)*

Yield 76%; m.p. >300 °C; silica gel TLC Rf 0.10 (TFA/MeOH/DCM 3/15/82% *v*/*v*); δ_H_ (400 MHz, DMSO-d_6_): 8.74 (s, 1H, Ar-*H*), 8.07 (d, *J* = 8.5 Hz, 2H, Ar-*H*), 7.91 (d, *J* = 8.5 Hz, 2H, Ar-*H*), 7.41 (s, 2H, exchange with D_2_O, SO_2_N*H*_2_), 4.56 (t, *J* = 6.1 Hz, 2H, C*H*_2_), 2.47 (t, *J* = 6.9 Hz, 2H, C*H*_2_), 2.18 (m, 2H, C*H*_2_). δ_C_ (100 MHz, DMSO-d_6_): 146.0, 144.0, 135.0, 127.4, 126.4, 123.7, 49.9, 49.0, 27.3; m/z (ESI negative): 345.2 [M]^−^.

*Sodium 3-(4-((4-sulfamoylphenoxy)methyl)-1H-1,2,3-triazol-1-yl)propane-1-sulfonate (***31***)*

Yield 70%; m.p. 290–293 °C; silica gel TLC Rf 0.10 (TFA/MeOH/DCM 3/15/82% *v*/*v*); δ_H_ (400 MHz, DMSO-d_6_): 8.28 (s, 1H, Ar-*H*), 7.77 (d, *J* = 8.58 Hz, 2H, Ar-*H*), 7.23 (s, 2H, exchange with D_2_O, SO_2_N*H*_2_), 7.21 (d, *J* = 8.58 Hz, 2H, Ar-*H*), 5.25 (s, 2H, C*H*_2_), 4.50 (t, *J* = 6.48 Hz, 2H, C*H*_2_), 2.39 (t, *J* = 6.48 Hz, 2H, C*H*_2_), 2.11 (m, 2H, C*H*_2_); δ_C_ (100 MHz, DMSO-d_6_): 161.4, 143.0, 137.5, 128.7, 125.9, 115.8, 62.4, 49.6, 48.9, 27.5; *m*/*z* (ESI negative): 375.2 [M].

*Sodium 3-(4-(((4-sulfamoylphenyl)amino)methyl)-1H-1,2,3-triazol-1-yl)propane-1-sulfonate (***32***)*

Yield 62%; m.p. >300 °C; silica gel TLC Rf 0.10 (TFA/MeOH/DCM 3/15/82% *v*/*v*); δ_H_ (400 MHz, DMSO-d_6_): 8.00 (s, 1H, Ar-*H*), 7.52 (d, *J* = 8.7 Hz, 2H, Ar-*H*), 6.94 (s, 2H, exchange with D_2_O, SO_2_N*H*_2_), 6.87 (t, *J* = 5.6 Hz, 1H, exchange with D_2_O, N*H*), 6.71 (d, *J* = 8.7 Hz, 2H, Ar-*H*), 4.45 (t, *J* = 6.2 Hz, 2H, C*H*_2_), 4.36 (d, *J* = 5.6 Hz, 2H, C*H*_2_), 2.38 (t, *J* = 7.5 Hz, 2H, C*H*_2_), 2.07 (m, 2H, C*H*_2_). δ_C_ (100 MHz, DMSO-d_6_): 152.0, 131.5, 128.3, 124.0, 112.1, 49.5, 49.0, 39.0, 27.5; *m*/*z* (ESI negative): 374.2 [M]^−^.

*Sodium 3-(4-((4-sulfamoylbenzamido)methyl)-1H-1,2,3-triazol-1-yl)propane-1-sulfonate (***33***)*

Yield 43%; m.p. >300 °C; silica gel TLC Rf 0.10 (TFA/MeOH/DCM 3/15/82% *v*/*v*); δ_H_ (400 MHz, DMSO-d_6_): 9.22 (t, *J* = 5.7 Hz, 1H, exchange with D_2_O, CON*H*), 8.06 (s, 1H, Ar-*H*), 8.03 (d, *J* = 8.5 Hz, 2H, Ar-*H*), 7.91 (d, *J* = 8.5 Hz, 2H, Ar-*H*), 7.50 (s, 2H, exchange with D_2_O, SO_2_N*H*_2_), 4.55 (d, *J* = 5.7 Hz, 2H, C*H*_2_), 4.45 (t, *J* = 6.6 Hz, 2H, C*H*_2_), 2.40 (t, *J* = 6.6 Hz, 2H, C*H*_2_), 2.09 (m, 2H, C*H*_2_). δ_C_ (100 MHz, DMSO-d_6_): 166.2, 147.3, 145.5, 138.1, 129.0, 126.6, 124.1, 49.5, 49.0, 36.0, 27.5; *m*/*z* (ESI negative): 402.3 [M]^−^.

*Sodium 3-(4-((3-(4-sulfamoylphenyl)ureido)methyl)-1H-1,2,3-triazol-1-yl)propane-1-sulfonate (***34***)*

Yield 71%; m.p. >300 °C; silica gel TLC Rf 0.10 (TFA/MeOH/DCM 3/15/82% *v*/*v*); δ_H_ (400 MHz, DMSO-d_6_): 9.35 (s, 1H, exchange with D_2_O, N*H*CON*H*), 7.97 (s, 1H, Ar-*H*), 7.69 (d, *J* = 8.2 Hz, 2H, Ar-*H*), 7.59 (d, J = 8.2 Hz, 2H, Ar-*H*), 7.20 (s, 3H, exchange with D_2_O, N*H*CON*H* + SO_2_N*H*_2_), 4.46 (t, *J* = 6.5 Hz, 2H, C*H*_2_), 4.36 (s, 2H, C*H*_2_), 2.52 (t, *J* = 6. Hz, 2H, C*H*_2_), 2.13 (m, 2H, C*H*_2_). δ_C_ (100 MHz, DMSO-d_6_): 155.8, 146.2, 144.7, 137.0, 127.7, 123.8, 117.9, 49.4, 49.3, 49.0, 35.9, 27.5; *m*/*z* (ESI negative): 417.3 [M]^−^.

*Sodium 3-(4-((3-(4-sulfamoylbenzyl)ureido)methyl)-1H-1,2,3-triazol-1-yl)propane-1-sulfonate (***35***)*

Yield 59%; m.p. 295–297 °C; silica gel TLC Rf 0.10 (TFA/MeOH/DCM 3/15/82% *v*/*v*); δ_H_ (400 MHz, DMSO-d_6_): 7.88 (s, 1H, Ar-*H*), 7.78 (d, *J* = 7.8 Hz, 2H, Ar-*H*), 7.42 (d, *J* = 7.8 Hz, 2H, Ar-*H*), 7.31 (s, 2H, exchange with D_2_O, SO_2_N*H*_2_), 6.59 (t, *J* = 5.5 Hz, 1H, exchange with D_2_O, N*H*CON*H*), 6.52 (t, *J* = 6.3 Hz, 1H, exchange with D_2_O, N*H*CON*H*), 4.44 (t, *J* = 6.3 Hz, 2H, C*H*_2_), 4.33–4.20 (m, 4H, 2 × C*H*_2_), 2.40 (t, *J* = 6.7 Hz, 2H, C*H*_2_), 2.09 (m, 2H, C*H*_2_), 1.83 (m, 2H, C*H*_2_). δ_C_ (100 MHz, DMSO-d_6_): 158.1, 146.0, 145.3, 142.5, 127.3, 125.7, 122.7, 49.9, 48.4, 48.3, 48.0, 25.0; *m*/*z* (ESI negative): 431.3 [M]^−^.

*Sodium 3-(4-((3-(4-sulfamoylphenethyl)ureido)methyl)-1H-1,2,3-triazol-1-yl)propane-1-sulfonate (***36***)*

Yield 43%; m.p. 217–219 °C; silica gel TLC Rf 0.10 (TFA/MeOH/DCM 3/15/82% *v*/*v*); δ_H_ (400 MHz, DMSO-d_6_): 7.87 (s, 1H, Ar-*H*), 7.76 (d, *J* = 7.9 Hz, 2H, Ar-*H*), 7.41 (d, *J* = 7.9 Hz, 2H, Ar-*H*), 7.31 (s, 2H, exchange with D_2_O, SO_2_N*H*_2_), 6.34 (t, *J* = 5.6 Hz, 1H, exchange with D_2_O, N*H*CON*H*), 5.99 (t, *J* = 6.1 Hz, 1H, exchange with D_2_O, N*H*CON*H*), 4.45 (t, *J* = 6.7 Hz, 2H, C*H*_2_), 4.24 (d, *J* = 5.6 Hz, 2H, C*H*_2_), 3.29 (q, *J* = 6.7, 6.1 Hz, 2H, C*H*_2_), 2.79 (t, *J* = 6.7 Hz, 2H, C*H*_2_), 2.39 (t, *J* = 6.7 Hz, 2H, C*H*_2_), 2.08 (m, 2H, C*H*_2_). δ_C_ (100 MHz, DMSO-d_6_): 158.9, 146.9, 145.0, 142.9, 130.2, 126.7, 123.5, 49.4, 49.0, 41.8, 36.8, 36.0, 27.5; *m*/*z* (ESI negative): 445.3 [M]^−^.

*Sodium 3-(4-((3-(4-sulfamoylphenyl)thioureido)methyl)-1H-1,2,3-triazol-1-yl)propane-1-sulfonate (***37***)*

Yield 52%; m.p. 230–232 °C; silica gel TLC Rf 0.10 (TFA/MeOH/DCM 3/15/82% *v*/*v*); δ_H_ (400 MHz, DMSO-d_6_): 9.98 (s, 1H, exchange with D_2_O, N*H*CSN*H*), 8.46 (t, *J* = 5.7 Hz, 1H, exchange with D_2_O, N*H*CSN*H*), 8.08 (s, 1H, Ar-*H*), 7.47 (q, *J* = 8.8 Hz, 4H, Ar-*H*), 7.28 (s, 2H, exchange with D_2_O, SO_2_N*H*_2_), 4.77 (d, *J* = 5.1 Hz, 2H, C*H*_2_), 4.48 (t, *J* = 6.6 Hz, 2H, C*H*_2_), 2.42 (t, *J* = 6.6 Hz, 2H, C*H*_2_), 2.10 (m, 2H, C*H*_2_). δ_C_ (100 MHz, DMSO-d_6_): 181.4, 144.6, 143.8, 139.5, 127.2, 124.2, 122.5, 49.5, 49.0, 27.5; *m*/*z* (ESI negative): 433.2 [M]^−^.

*Sodium 3-(4-((4-(3-(4-sulfamoylphenyl)ureido)phenoxy)methyl)-1H-1,2,3-triazol-1-yl)propane-1-sulfonate (***38***)*

Yield 36%; m.p. >300 °C; silica gel TLC Rf 0.10 (TFA/MeOH/DCM 3/15/82% *v*/*v*); δ_H_ (400 MHz, DMSO-d_6_): 9.85 (s, 1H, exchange with D_2_O, N*H*CON*H*), 9.47 (s, 1H, exchange with D_2_O, N*H*CON*H*), 8.23 (s, 1H, Ar-*H*), 7.72 (d, *J* = 8.3 Hz, 2H, Ar-*H*), 7.65 (d, *J* = 8.3 Hz 2H, Ar-*H*), 7.43 (d, *J* = 8.8 Hz, 2H, Ar-*H*), 7.20 (s, 2H, exchange with D_2_O, SO_2_N*H*_2_), 6.99 (d, *J* = 8.8 Hz, 2H, Ar-*H*), 5.10 (s, 2H, C*H*_2_), 4.50 (t, *J* = 6.7 Hz, 2H, C*H*_2_), 2.41 (t, *J* = 6.8 H, 2H, C*H*_2_), 2.11 (m, 2H, C*H*_2_). δ_C_ (100 MHz, DMSO-d_6_): 154.3, 153.8, 144.7, 143.8, 137.2, 134.3, 127.7, 125.5, 120.9, 118.1, 115.9, 62.4, 49.6, 49.0, 27.5; *m*/*z* (ESI negative): 509.3 [M]^−^.

*Sodium 3-(4-((2-acetamido-4-sulfamoylphenoxy)methyl)-1H-1,2,3-triazol-1-yl)propane-1-sulfonate (***39***)*

Yield 39%; m.p. >300 °C; silica gel TLC Rf 0.10 (TFA/MeOH/DCM 3/15/82% *v*/*v*); δ_H_ (400 MHz, DMSO-d_6_): 9.19 (s, 1H, exchange with D_2_O, CON*H*), 8.51 (s, 1H, Ar-*H*), 8.30 (s, 1H, Ar-*H*), 7.55 (d, *J* = 8.4 Hz, 1H, Ar-*H*), 7.43 (d, *J* = 8.4 Hz, 1H, Ar-*H*), 7.24 (s, 2H, exchange with D_2_O, SO_2_N*H*_2_), 5.36 (s, 2H, C*H*_2_), 4.52 (t, *J* = 6.7 Hz, 2H, C*H*_2_), 2.43 (m, 6H, 3 × C*H*_2_), 2.12 (s, 3H, C*H*_3_). δ_C_ (100 MHz, DMSO-d_6_): 173.5, 163.4, 143.1, 137.3, 128.8, 125.9, 123.1, 120.6, 113.7, 63.4, 49.6, 48.9, 31.8, 30.3, 10.7; *m*/*z* (ESI negative): 432.2 [M]^−^.

*Sodium 3-(4-((2-propionamido-4-sulfamoylphenoxy)methyl)-1H-1,2,3-triazol-1-yl)propane-1-sulfonate (***40***)*

Yield 41%; m.p. >300 °C; silica gel TLC Rf 0.10 (TFA/MeOH/DCM 3/15/82% *v*/*v*); δ_H_ (400 MHz, DMSO-d_6_): 9.30 (s, 1H, exchange with D_2_O, CON*H*), 8.47 (s, 1H, Ar-*H*), 8.30 (s, 1H, Ar-*H*), 7.54 (d, *J* = 8.1 Hz, 1H, Ar-*H*), 7.42 (d, *J* = 8.1 Hz, 1H, Ar-*H*), 7.24 (s, 2H, exchange with D_2_O, SO_2_N*H*_2_), 5.35 (s, 2H, C*H*_2_), 4.50 (t, *J* = 6.9 Hz, 2H, C*H*_2_), 2.43 (t, *J* = 6.8 Hz, 2H, C*H*_2_), 2.12 (s, 3H, C*H*_3_), 2.10 (m, 2H, C*H*_2_). δ_C_ (100 MHz, DMSO-d_6_): 170.2, 163.5, 151.9, 143.3, 137.7, 129.0, 126.2, 123.2, 121.1, 114.2, 63.8, 49.8, 49.3, 27.6, 25.4; *m*/*z* (ESI negative): 446.3 [M]^−^.

*Sodium 3-(4-(4-sulfamoylphenyl)-1H-1,2,3-triazol-1-yl)butane-1-sulfonate (***41***)*

Yield 81%; m.p. >300 °C; silica gel TLC Rf 0.10 (TFA/MeOH/DCM 3/15/82% *v*/*v*); δ_H_ (400 MHz, DMSO-d_6_): 8.78 (s, 1H, Ar-*H*), 8.05 (d, *J* = 8.5 Hz, 2H, Ar-*H*), 7.91 (d, *J* = 8.5 Hz, 2H, Ar-*H*), 7.44 (s, 2H, exchange with D_2_O, SO_2_N*H*_2_), 4.43 (t, *J* = 6.3 Hz, 2H, C*H*_2_), 1.96 (t, *J* = 6.6 Hz, 2H, C*H*_2_), 1.66 (m, 2H, C*H*_2_), 1.57 (m, 2H, C*H*_2_). δ_C_ (100 MHz, DMSO-d_6_): 146.0, 144.1, 135.0, 127.4, 126.3, 123.5, 51.6, 50.5, 29.9, 23.2; *m*/*z* (ESI negative): 359.2 [M]^−^.

*Sodium 3-(4-((4-sulfamoylphenoxy)methyl)-1H-1,2,3-triazol-1-yl)butane-1-sulfonate (***42***)*

Yield 68%; m.p. >300 °C; silica gel TLC Rf 0.10 (TFA/MeOH/DCM 3/15/82% *v*/*v*); δ_H_ (400 MHz, DMSO-d_6_): 8.29 (s, 1H, Ar-*H*), 7.77 (d, *J* = 8.4 Hz, 2H, Ar-*H*), 7.24 (s, 2H, exchange with D_2_O, SO_2_N*H*_2_), 7.22 (d, *J* = 8.4 Hz, 2H, Ar-*H*), 5.25 (s, 2H, C*H*_2_), 4.38 (t, *J* = 6.7 Hz, 2H, C*H*_2_), 2.45 (t, *J* = 7.6 Hz, 2H, C*H*_2_), 1.90 (m, 2H, C*H*_2_), 1.54 (m, 2H, C*H*_2_). δ_C_ (100 MHz, DMSO-d_6_): 161.4, 143.0, 137.5, 128.7, 125.7, 115.8, 62.5, 51.7, 51.6, 50.3, 30.0, 28.6, 23.4, 23.1; *m*/*z* (ESI negative): 389.1 [M]^−^.

*Sodium 3-(4-(((4-sulfamoylphenyl)amino)methyl)-1H-1,2,3-triazol-1-yl)butane-1-sulfonate* (**43**)

Yield 51%; m.p. 267–269 °C; silica gel TLC Rf 0.10 (TFA/MeOH/DCM 3/15/82% *v*/*v*); δ_H_ (400 MHz, DMSO-d_6_): 8.01 (s, 1H, Ar-*H*), 7.53 (d, *J* = 8.2 Hz, 2H, Ar-*H*), 6.94 (s, 2H, exchange with D_2_O, SO_2_N*H*_2_), 6.89 (t, *J* = 5.5 Hz, 1H, exchange with D_2_O, N*H*), 6.71 (d, *J* = 8.2 Hz, 2H, Ar-*H*), 4.34 (m, 4H, 2 × C*H*_2_), 2.43 (t, *J* = 7.1 Hz, 2H, C*H*_2_), 1.87 (m, 2H, C*H*_2_), 1.52 (m, 2H, C*H*_2_). δ_C_ (100 MHz, DMSO-d_6_): 152.0, 145.7, 131.5, 128.3, 123.8, 112.1, 51.6, 50.2, 39.1, 30.1, 23.2; *m*/*z* (ESI negative): 388.2 [M]^−^.

*Sodium 3-(4-((4-sulfamoylbenzamido)methyl)-1H-1,2,3-triazol-1-yl)butane-1-sulfonate (***44***)*

Yield 59%; m.p. >300 °C; silica gel TLC Rf 0.10 (TFA/MeOH/DCM 3/15/82% *v*/*v*); δ_H_ (400 MHz, DMSO-d_6_): 9.26 (t, *J* = 5.4 Hz, 1H, exchange with D_2_O, CON*H*), 8.05 (d, *J* = 7.8 Hz, 2H, Ar-*H*), 8.01 (s, 1H, Ar-*H*), 7.91 (d, *J* = 7.8 Hz, 2H, Ar-*H*), 7.50 (s, 2H, exchange with D_2_O, SO_2_N*H*_2_), 4.55 (d, *J* = 5.4 Hz, 2H, C*H*_2_), 4.33 (t, *J* = 6.8 Hz, 2H, C*H*_2_), 2.45 (t, *J* = 7.1 Hz, 2H, C*H*_2_), 1.88 (m, 2H, C*H*_2_), 1.54 (m, 2H, C*H*_2_). δ_C_ (100 MHz, DMSO-d_6_): 166.2, 147.3, 145.6, 138.0, 129.0, 126.6, 123.9, 51.6, 50.2, 36.0, 30.1, 23.2; *m*/*z* (ESI negative): 416.2 [M]^−^.

*Sodium 3-(4-((3-(4-sulfamoylphenyl)ureido)methyl)-1H-1,2,3-triazol-1-yl)butane-1-sulfonate (***45***)*

Yield 62%; m.p. >300 °C; silica gel TLC Rf 0.10 (TFA/MeOH/DCM 3/15/82% *v*/*v*); δ_H_ (400 MHz, DMSO-d_6_): 9.14 (s, 1H, exchange with D_2_O, N*H*CON*H*), 8.00 (s, 1H, Ar-*H*), 7.69 (d, *J* = 8.5 Hz, 2H, Ar-*H*), 7.58 (d, *J* = 8.5 Hz, 2H, Ar-*H*), 7.18 (s, 2H, exchange with D_2_O, SO_2_N*H*_2_), 6.91 (t, *J* = 5.2 Hz, 1H, exchange with D_2_O, N*H*CON*H*), 4.36 (m, 4H, 2 × C*H*_2_), 2.42 (t, *J* = 6.7 Hz, 2H, C*H*_2_), 1.88 (m, 2H, C*H*_2_), 1.54 (m, 2H, C*H*_2_). δ_C_ (100 MHz, DMSO-d_6_): 155.8, 146.2, 144.7, 137.0, 127.7, 123.6, 117.8, 51.6, 50.2, 49.6, 30.1, 23.2; *m*/*z* (ESI negative): 431.2 [M]^−^.

*Sodium 3-(4-((3-(4-sulfamoylbenzyl)ureido)methyl)-1H-1,2,3-triazol-1-yl)butane-1-sulfonate* (**46**)

Yield 42%; m.p. >300 °C; silica gel TLC Rf 0.10 (TFA/MeOH/DCM 3/15/82% *v*/*v*); δ_H_ (400 MHz, DMSO-d_6_): 7.90 (s, 1H, Ar-*H*), 7.78 (d, *J* = 8.3 Hz, 2H, Ar-*H*), 7.42 (d, *J* = 8.3 Hz, 2H, Ar-*H*), 7.32 (s, 2H, echange with D_2_O, SO_2_N*H*_2_), 6.59 (t, *J* = 6.5 Hz, 1H, exchange with D_2_O, N*H*CON*H*), 6.54 (t, *J* = 5.8 Hz, 1H, exchange with D_2_O, N*H*CON*H*), 4.39–4.22 (m, 6H, 3 × CH*2*), 2.44 (t, *J* = 6.5 Hz, 2H, C*H*_2_), 1.87 (m, 2H, C*H*_2_), 1.54 (m, 2H, C*H*_2_). δ_C_ (100 MHz, DMSO-d_6_): 159.0, 146.8, 146.2, 143.4, 128.2, 126.6, 123.4, 51.7, 50.2, 43.6, 36.2, 30.1, 23.2; *m*/*z* (ESI negative): 445.3 [M]^−^.

*Sodium 3-(4-((3-(4-sulfamoylphenethyl)ureido)methyl)-1H-1,2,3-triazol-1-yl)butane-1-sulfonate* (**47**)

Yield 20%; m.p. >300 °C; silica gel TLC Rf 0.10 (TFA/MeOH/DCM 3/15/82% *v*/*v*); δ_H_ (400 MHz, DMSO-d_6_): 7.88 (s, 1H, Ar-*H*), 7.77 (d, *J* = 7.9 Hz, 2H, Ar-*H*), 7.42 (d, *J* = 7.9 Hz, 2H, Ar-*H*), 7.32 (s, 2H, echange with D_2_O, SO_2_N*H*_2_), 6.33 (t, *J* = 5.4 Hz, 1H, exchange with D_2_O, N*H*CON*H*), 6.05 (t, *J* = 6.1 Hz, 1H, exchange with D_2_O, N*H*CON*H*), 4.33 (t, *J* = 6.9 Hz, 2H, C*H*_2_), 4.24 (d, *J* = 5.4 Hz, 2h, C*H*_2_), 3.79 (t, *J* = 6.7 Hz, 2H, C*H*_2_), 3.28 (q, *J* = 6.5, 6.1 Hz, 2H, C*H*_2_), 2.78 (t, *J* = 6.5 Hz, 2H, C*H*_2_), 1.59 (m, 2H, C*H*_2_), 1.33 (m, 2H, C*H*_2_). δ_C_ (100 MHz, DMSO-d_6_): 158.8, 146.9, 145.0, 143.0, 130.1, 126.7, 123.4, 51.7, 50.2, 41.7, 36.8, 36.0, 24.1, 20.2; *m*/*z* (ESI negative): 459.3 [M]^−^.

*Sodium 3-(4-((3-(4-sulfamoylphenyl)thioureido)methyl)-1H-1,2,3-triazol-1-yl)butane-1-sulfonate* (**48**)

Yield 34%; m.p. 240–242 °C; silica gel TLC Rf 0.10 (TFA/MeOH/DCM 3/15/82% *v*/*v*); δ_H_ (400 MHz, DMSO-d_6_): 10.0 (s, 1H, exchange with D_2_O, N*H*CSN*H*), 8.50 (t, *J* = 4.2 Hz, 1H, exchange with D_2_O, N*H*CSN*H*), 8.09 (s, 1H, Ar-*H*), 7.76 (s, 4H, Ar-*H*), 7.30 (s, 2H, exchange with D_2_O, SO_2_N*H*_2_), 4.77 (d, *J* = 4.2 Hz, 2H, C*H*_2_), 4.37 (t, *J* = 6.8 Hz, 2H, C*H*_2_), 2.43 (t, *J* = 7.4 Hz, 2H, C*H*_2_), 1.89 (m, 2H, C*H*_2_), 1.55 (m, 2H, C*H*_2_). δ_C_ (100 MHz, DMSO-d_6_): 181.5, 144.6, 143.9, 139.4, 127.2, 124.1, 122.3, 51.6, 50.3, 42.2, 30.1, 23.1; *m*/*z* (ESI negative): 447.2 [M]^−^.

*Sodium 3-(4-((4-(3-(4-sulfamoylphenyl)ureido)phenoxy)methyl)-1H-1,2,3-triazol-1-yl)butane-1-sulfonate* (**49**)

Yield 19%; m.p. >300 °C; silica gel TLC Rf 0.10 (TFA/MeOH/DCM 3/15/82% *v*/*v*); δ_H_ (400 MHz, DMSO-d_6_): 10.41 (s, 1H, exchange with D_2_O, N*H*CON*H*), 10.0 (s, 1H, exchange with D_2_O, N*H*CON*H*), 8.24 (s, 1H, Ar-*H*), 7.70 (q, *J* = 8.3 Hz, 4H, Ar-*H*), 7.46 (d, *J* = 8.2 Hz, 2H, Ar-*H*), 7.19 (s, 2H, exchange with D_2_O, SO_2_N*H*_2_), 6.98 (d, *J* = 8.3 Hz, 2H, Ar-*H*), 5.10 (s, 2H, C*H*_2_), 4.38 (t, *J* = 6.7 Hz, 2H, C*H*_2_), 2.46 (t, *J* = 6.6 Hz, 2H, C*H*_2_), 1.90 (m, 2H, C*H*_2_), 1.55 (m, 2H, C*H*_2_). δ_C_ (100 MHz, DMSO-d_6_): 154.2, 154.0, 145.1, 143.8, 137.1, 134.6, 127.7, 125.5, 120.9, 118.1, 115.9, 51.6, 50.3, 30.1, 23.5, 23.2; *m*/*z* (ESI negative): 523.3 [M]^−^.

*Sodium 3-(4-((2-acetamido-4-sulfamoylphenoxy)methyl)-1H-1,2,3-triazol-1-yl)butane-1-sulfonate* (**50**)

Yield 18%; m.p. >300 °C; silica gel TLC Rf 0.10 (TFA/MeOH/DCM 3/15/82% *v*/*v*); δ_H_ (400 MHz, DMSO-d_6_): 9.19 (s, 1H, exchange with D_2_O, CON*H*), 8.51 (s, 1H, Ar-*H*), 8.30 (s, 1H, Ar-*H*), 7.55 (d, *J* = 8.6 Hz, 1H, Ar-*H*), 7.42 (d, *J* = 8.6 Hz, 1H, Ar-*H*), 7.24 (s, 2H, exchange with D_2_O, SO_2_N*H*_2_), 5.36 (s, 2H, C*H*_2_), 4.40 (t, *J* = 6.3 Hz, 2H, C*H*_2_), 2.46 (t, *J* = 7.1 Hz, 4H, 2 × C*H*_2_), 1.92 (m, 2H, C*H*_2_), 1.56 (m, 2H, C*H*_2_). δ_C_ (100 MHz, DMSO-d_6_): 173.6, 170.2, 143.5, 137.7, 129.0, 125.8, 123.2, 119.1, 114.2, 63.8, 51.7, 50.4, 30.1, 30.3, 30.1, 10.8; *m*/*z* (ESI negative): 446.2 [M]^−^.

*Sodium 3-(4-((2-propionamido-4-sulfamoylphenoxy)methyl)-1H-1,2,3-triazol-1-yl)butane-1-sulfonate* (**51**)

Yield 23%; m.p. >300 °C; silica gel TLC Rf 0.10 (TFA/MeOH/DCM 3/15/82% *v*/*v*); δ_H_ (400 MHz, DMSO-d_6_): 9.30 (s, 1H, exchange with D_2_O, CON*H*), 8.47 (s, 1H, Ar-*H*), 8.30 (s, 1H, Ar-*H*), 7.54 (d, *J* = 8.1 Hz, 1H, Ar-*H*), 7.41 (d, *J* = 8.1 Hz, 1H, Ar-*H*), 7.24 (s, 2H, exchange with D_2_O, SO_2_N*H*_2_), 5.35 (s, 2H, C*H*_2_), 4.36 (t, *J* = 6.3 Hz, 2H, C*H*_2_), 2.46 (t, *J* = 7.1 Hz, 2H, C*H*_2_), 2.13 (s, 3H, C*H*_3_), 1.91 (m, 2H, C*H*_2_), 1.55 (m, 2H, C*H*_2_). δ_C_ (100 MHz, DMSO-d_6_): 173.5, 163.4, 151.5, 143.1, 137.3, 128.8, 125.9, 123.1, 120.6, 113.7, 63.4, 49.6, 48.9, 31.8, 30.3, 27.4; *m*/*z* (ESI negative): 460.3 [M]^−^.

### 3.2. Carbonic Anhydrase Inhibition

An Applied Photophysics stopped-flow instrument was used for assaying the CA catalyzed CO_2_ hydration activity [58]. Phenol red (at a concentration of 0.2 mM) was used as an indicator, working at the absorbance maximum of 557 nm, with 20 mM HEPES (pH 7.5) as buffer, and 20 mM Na_2_SO_4_ (for maintaining constant the ionic strength), following the initial rates of the CA-catalyzed CO_2_ hydration reaction for a period of 10–100 s. The CO_2_ concentrations ranged from 1.7 to 17 mM for the determination of the kinetic parameters and inhibition constants. For each inhibitor, at least six traces of the initial 5–10% of the reaction were used for determining the initial velocity. The uncatalyzed rates were determined in the same manner and subtracted from the total observed rates. Stock solutions of inhibitor (0.1 mM) were prepared in distilled–deionized water, and dilutions up to 0.01 nM were done thereafter with the assay buffer. Inhibitor and enzyme solutions were preincubated together for 45 min at room temperature prior to assay in order to allow for the formation of the E–I complex. The inhibition constants were obtained by non-linear least-squares methods using PRISM 3 and the Cheng–Prusoff equation, as reported earlier [31], representing the mean from at least three different determinations. The enzyme concentrations were in the range of 5–16 nM. All CA isoforms were recombinant ones obtained in-house, as reported earlier [32].

### 3.3. Caco-2 Cells

The colorectal adenocarcinoma cell line Caco-2 was purchased from American Tissue Type Culture Collection (Manassas, VA, USA) and cultured in Dulbecco’s modified Eagle’s medium (DMEM) (Thermo Fisher Scientific, Rodano, Milan, Italy) with 20% fetal bovine serum (FBS) (Thermo Fisher Scientific, Rodano, Milan, Italy) and 100 U/mL penicillin–streptomycin (Thermo Fisher Scientific, Rodano, Milan, Italy) in 5% CO_2_ at 37 °C. Cell viability was determined by MTS after 2 h of exposure to the aliphatic sulfonate CAIs at 50 µM concentration, as previously described [59]. 

To test the cellular uptake of the sulfonate CAIs, we seeded Caco-2 cells in 12-well plates at a density of 1 × 10^5^ cells per well. After 24 h, cells were exposed to the novel compounds, 50 µM in DMEM, with 20% FBS without phenol red (Thermo Fisher Scientific, Rodano, Milan, Italy), for 2 h at 37 °C. After that, culture media and cell lysates were collected for FIA-MS/MS analyses.

### 3.4. FIA-MS/MS Method

Acetonitrile and acetone (Chromasolv) were purchased by Sigma-Aldrich (Milan, Italy). The mQ water, 18 MΩ cm, was obtained from Millipore’s Simplicity system (Milan, Italy). The LC–MS/MS analysis was carried out using a Varian 500-MS ion trap system (Palo Alto, CA, USA) equipped by two Prostar 210 pumps, a Prostar 410 autosampler, and an electrospray source (ESI). Raw data were collected and processed by Varian Workstation version 6.8 software. The ESI source was operated in negative ion mode, using the following setting: −5 kV needle, 45 psi nebulizing gas, 600 V shield, and 15 psi drying gas at 280 °C. The analyses were acquired in products ion scan (PIS) using 50 ms of excitation time and the precursor ions, excitation voltages (EV), and product ions used as quantifier ion are reported in Table S1.

A series of energy-resolved tandem mass spectrometry (ERMS) experiments were performed to study the fragmentation of molecular species of each analyte and build its breakdown curves. The ERMS experiments were carried out by introducing a 1 µg mL^−1^ solution of each analyte, via syringe pump at 10 µL min^−1^; the protonated molecule was isolated, and the abundance of PIS were monitored. The PIS spectra were acquired in the range from *m*/*z* 50 to 650, with a scan time of 600 ms; helium was used as collisional gas, and the EV was increased stepwise in the range 0–1.5 V. The breakdown curves were built using the relative intensity values of each signal present in the MS/MS spectra; they were obtained by averaging 15–20 scans for each EV (Figures S1–S6). 

The mobile phase used in the isocratic flow injection analysis (FIA) consisted of mQ water/acetonitrile 50:50 (*v*/*v*). The preparation of the samples consisted of the addition of acetonitrile to the culture cell medium in microcentrifuge tubes and then centrifuging the mixture (room temperature for 5 min at 8000 rpm). The supernatant was then collected and transferred in autosampler vials and analyzed with FIA-MS/MS method. In order to evaluate the limit of detection (LoD) of each analyte by using FIA-MS/MS method, we carried out a signal-to-noise (S/N) approach [60]. By the analysis of low concentration solutions of each sulfonate CAIs, the results showed that the LoD (S/N ≥ 3) of the determination was 2% of the initial concentration (spiked solution).

## 4. Conclusions

We report here a new drug design strategy for obtaining membrane-impermeant CAIs for selectively targeting the tumor-associated, membrane-bound hCAs IX and XII over off-target cytosolic isoforms. Unlike previously investigated positively charged pyridinium-based CAIs, aliphatic (propyl and butyl) sulfonic acid tails were included for the first time onto a benzenesulfonamide scaffold by a common 1,2,3-triazole linker and different types of spacers such as ether, amine, amide, urea, and thiourea. In fact, the deprotonated sulfonate form of these derivatives is permanently charged at physiological pH and suitable for increasing the CAI hydrophilicity up to hindering membrane permeability. Twenty such derivatives were tested for their inhibition of a panel of CA isoforms composed of the off-target ubiquitous hCAs I and II, and the target membrane-associated hCAs IV, IX, and XII. Most sulfonate CAIs induced a potent inhibition of hCAs II, IX, and XII up to a low nanomolar K_I_ range. Despite the tumor-associated hCAs being effectively inhibited by most produced derivatives, only a few compounds showed significant *SI* values against hCAs IX and XII over hCA II. On the contrary, all compounds demonstrated a preferential hCA IX and XII inhibitory efficacy over the main-off target hCA I. Derivative **43** from the butylsulfonate subset stood out as the most pharmacodynamically selective CAI against hCA IX and XII over off-target isoforms. According to the drug design schedule, a subset of representative derivatives was assessed for their cell membrane permeability using Caco-2 cells and a developed FIA-MS/MS method. All tested sulfonate CAIs demonstrated a complete membrane impermeability (≥98%) that validated such negatively charged moiety as a suitable tool for producing the selective targeting of the tumor-associated CAs in vivo. The most active such compounds will be assessed for their antiproliferative action against hCA IX/XII-overexpressing cancer cell lines (e.g., A549, PC-3, and HCT-116) in normoxic and hypoxic conditions. Moreover, their action on a number of pro/anti-apoptotic markers will be evaluated for identifying promising leads for the development of new anticancer therapies.

## Figures and Tables

**Table 1:** Inhibition data of CA isoforms I, II, IV, IX, and XII with sulfonamides **30**–**51** and the standard sulfonamide inhibitor **AAZ** by a stopped flow CO_2_ hydrase assay [58].

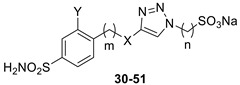									
Cmpd	KI (nM)a								
	Y	m	X	n	CA I	CA II	CA IV	CA IX	CA XII
**30**	H	0	-	3	83.5	3.2	429.0	35.9	6.7
**31**	H	0	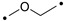	3	82.7	0.9	38.3	44.4	47.7
**32**	H	0	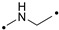	3	460.4	4.2	43.1	459.4	433.1
**33**	H	0	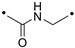	3	457.8	4.3	56.3	3.2	9.5
**34**	H	0	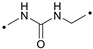	3	622.3	7.4	63.5	68.2	67.6
**35**	H	1	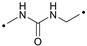	3	806.8	138.6	50.2	71.8	16.4
**36**	H	2	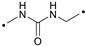	3	76.8	43.1	869.7	282.0	8.7
**37**	H	0	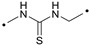	3	79.9	21.4	78.1	51.2	9.4
**38**	H	0	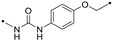	3	51.6	5.4	36.7	54.4	49.0
**39**	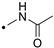	0	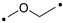	3	>10,000	63.4	244.0	242.0	18.7
**40**	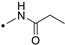	0	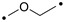	3	9160	6.6	322.1	54.8	47.1
**41**	H	0	-	4	149.5	18.8	407.9	8.6	43.1
**42**	H	0	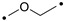	4	96.8	29.5	750.0	60.2	23.1
**43**	H	0	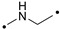	4	2909	363.8	882.8	79.9	3.4
**44**	H	0	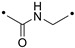	4	168.4	22.5	651.3	50.7	32.2
**45**	H	0	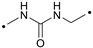	4	496.2	41.1	149.7	371.6	33.6
**46**	H	1	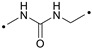	4	650.9	331.8	635.8	132.7	31.3
**47**	H	2	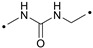	4	82.3	41.3	473.11	68.1	64.0
**48**	H	0	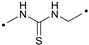	4	62.4	5.8	557.0	8.8	31.5
**49**	H	0	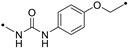	4	91.9	18.8	4540	32.2	53.2
**50**	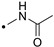	0	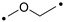	4	7687	263.3	3564	82.5	40.6
**51**	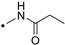	0	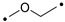	4	3650	39.9	>10,000	235.7	7.3
**AAZ**	-	-	-	-	250	12.0	74	25.0	5.7

Mean from 3 different assays by a stopped flow technique (errors in the range of ±5–10% of the reported values).

**Table 2:** Selectivity index (*SI*) calculated for hCA IX and XII over off-targets hCA I and II as ratio between K_I_ values.

Cmpd	CA I/CA IX	CA I/CA XII	CA II/CA IX	CA II/CA XII
**30**	2.3	12	0.09	0.48
**31**	1.8	1.7	0.02	0.02
**32**	1.0	1.1	0.01	0.01
**33**	143	48.2	1.3	0.45
**34**	9.1	9.2	0.11	0.11
**35**	11	49	1.9	8.4
**36**	0.3	8.8	0.15	4.9
**37**	1.	8.5	0.42	2.2
**38**	0.95	1.1	0.10	0.11
**39**	>41	>534	0.26	3.4
**40**	167	194	0.12	0.14
**41**	17	3.4	2.2	0.44
**42**	1.6	4.1	0.49	1.2
**43**	36	855	4.5	107
**44**	3.3	5.2	0.44	0.70
**45**	1.3	14	0.11	1.2
**46**	1.8	1.7	0.02	0.02
**47**	1.0	1.1	0.01	0.01
**48**	143	48.2	1.3	0.45
**49**	9.1	9.2	0.11	0.11
**50**	11	49	1.9	8.4
**51**	0.3	8.8	0.15	4.9
**AAZ**	10	43	0.48	2.1

## Data Availability

Not applicable.

## Supplementary Materials

The following are available online at https://www.mdpi.com/article/10.3390/ijms23010461/s1.
