# Supplementary material for: Aromatic Sulfonamides including a Sulfonic Acid Tail: New Membrane Impermeant Carbonic Anhydrase Inhibitors for Targeting Selectively the Cancer-Associated Isoforms
Source: Int J Mol Sci. 2021 Dec 31;23(1):461. doi: 10.3390/ijms23010461 (PMC8745330; doi:10.3390/ijms23010461)
Supplement: Supplementary file 1 [file ijms-23-00461-s001.zip › ijms-1506264-supplementary.pdf]

Supporting information

Aromatic sulfonamides including a sulfonic acid tail: new membrane impermeant carbonic anhydrase inhibitors for targeting selectively the cancer-associated isoforms.

Simone Giovannuzzi [1], Mario D'Ambrosio [2], Cristina Luceri [2], Marco Pallecchi [1], Gianluca Bartolucci [1], Alessio Nocentini [1]* and Claudiu T. Supuran [1]

[1] Department of NEUROFARBA, Section of Pharmaceutical and Nutraceutical Sciences, University of Florence, Polo Scientifico, Via U. Schiff 6, 50019 Sesto Fiorentino, Firenze, Italy.

[2] University of Florence, Department of NEUROFARBA, Section of Pharmacology and Toxicology, Viale Gaetano Pieraccini 6, 50100, Florence, Italy.

Table S1. MS/MS parameters

Compound	Precursor ion (m/z)	Quantifier ion (m/z) [EV (V)]
**33**	402	280 [1,0]
**34**	417	219 [1,0]
**38**	509	311 [1,0]
**39**	432	367 [1,0]
**41**	359	223 [0,8]
**43**	388	252 [1,2]

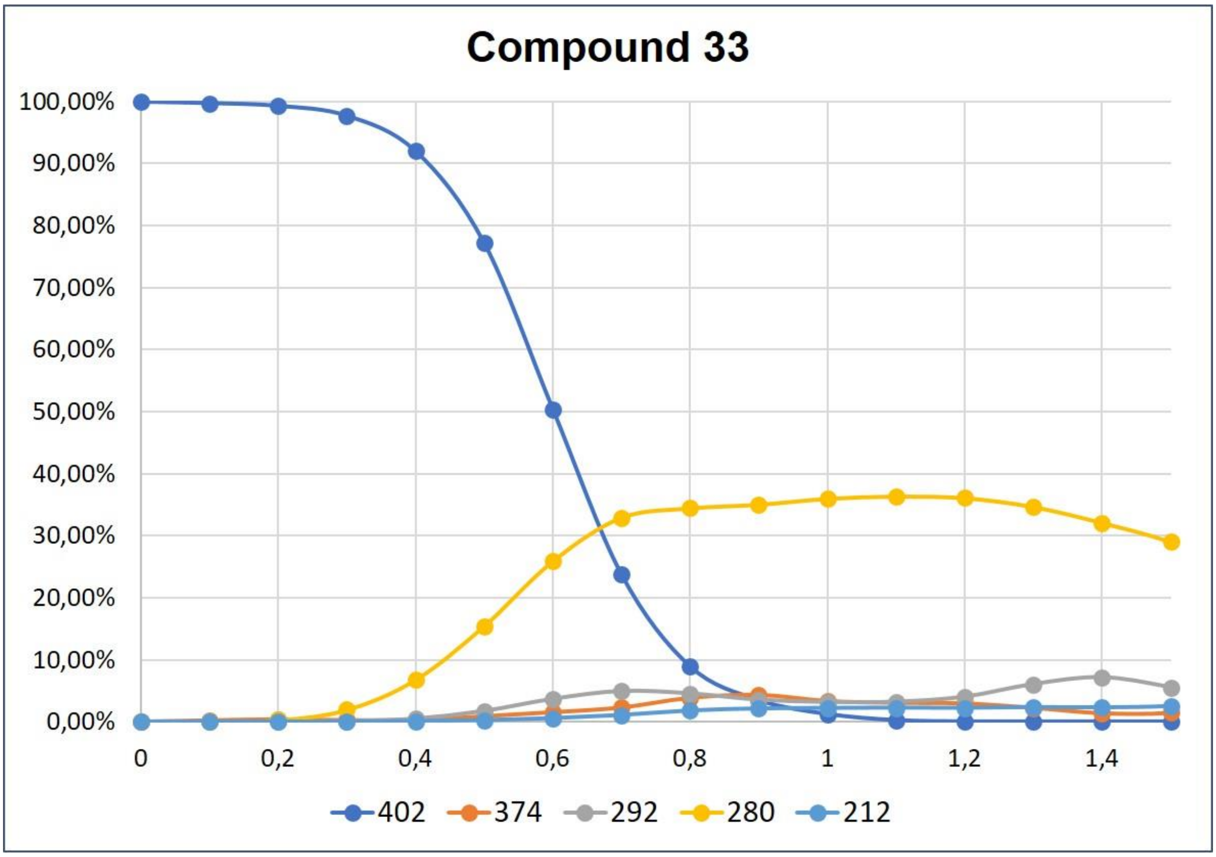

Figure S1. Breakdown curves of compound **33**.

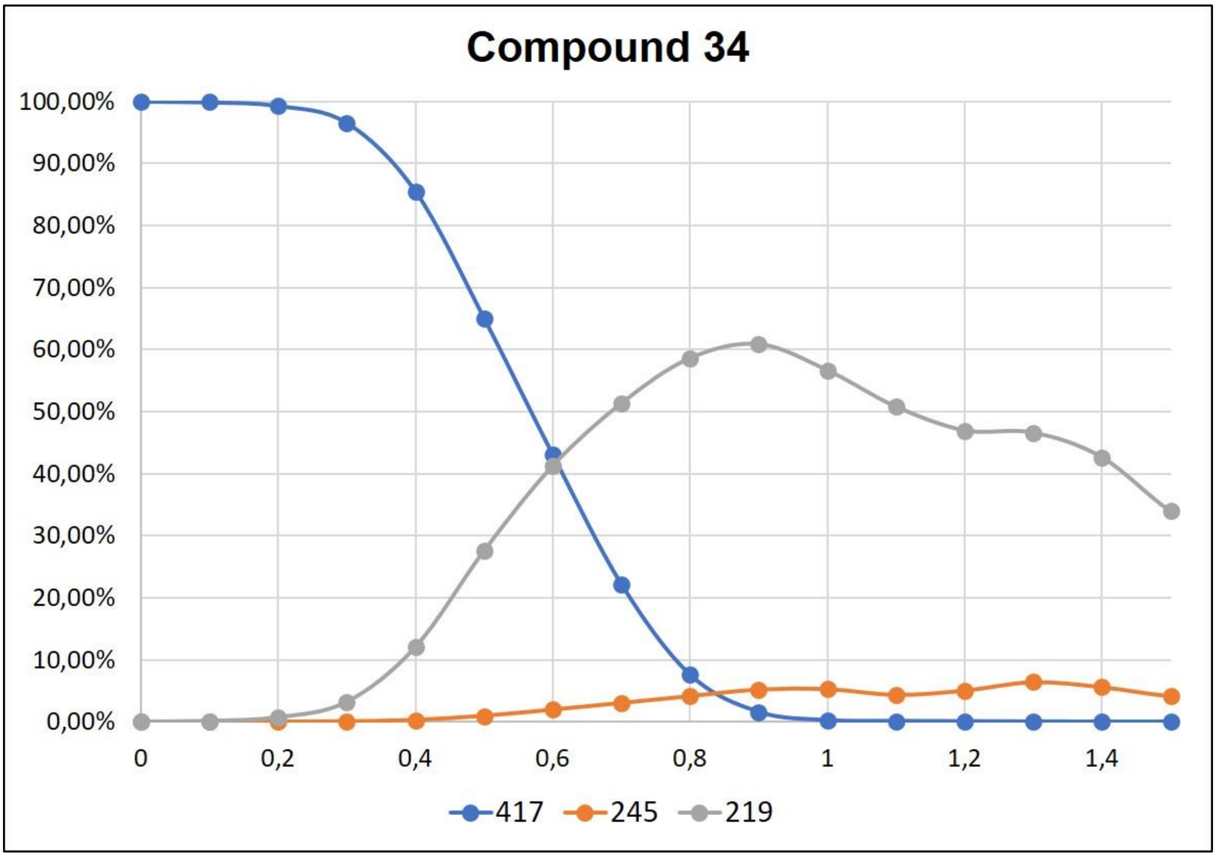

Figure S2. Breakdown curves of compound **34**.

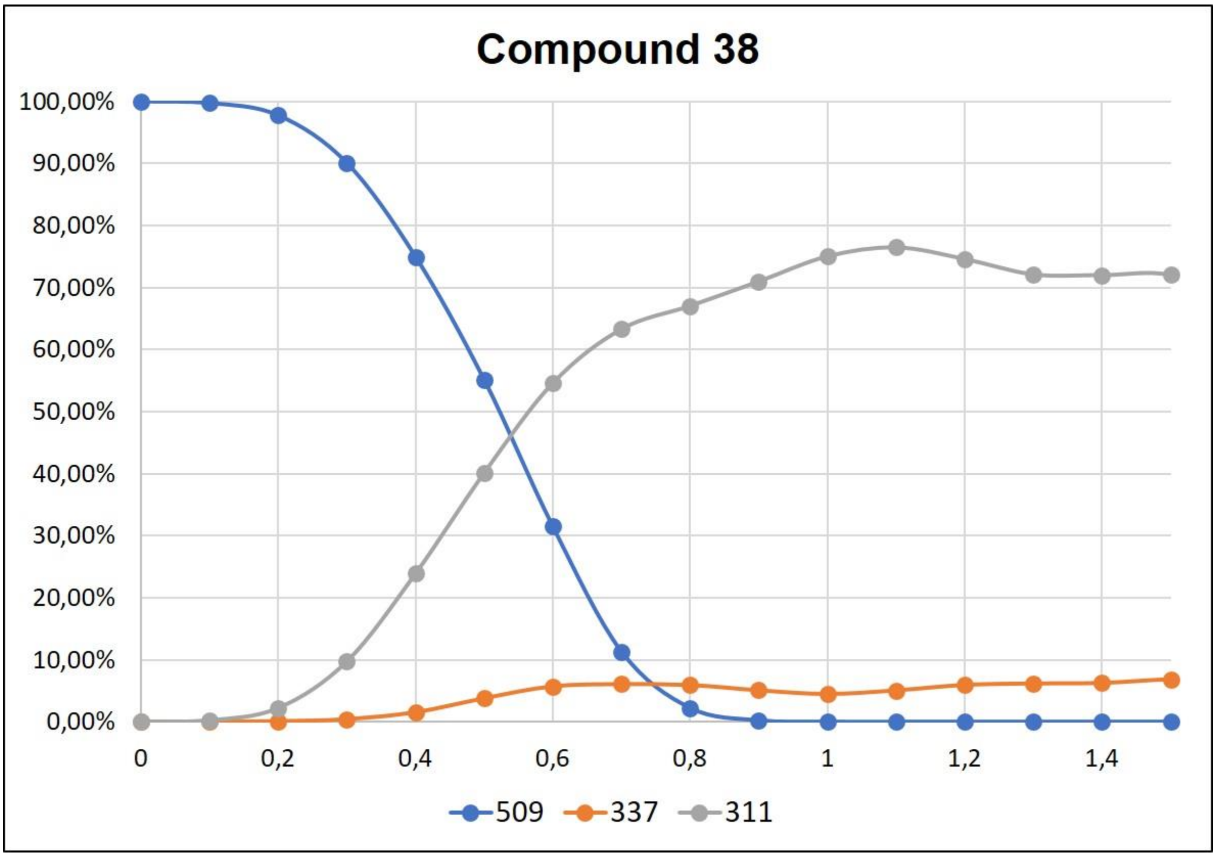

Figure S3. Breakdown curves of compound **38**.

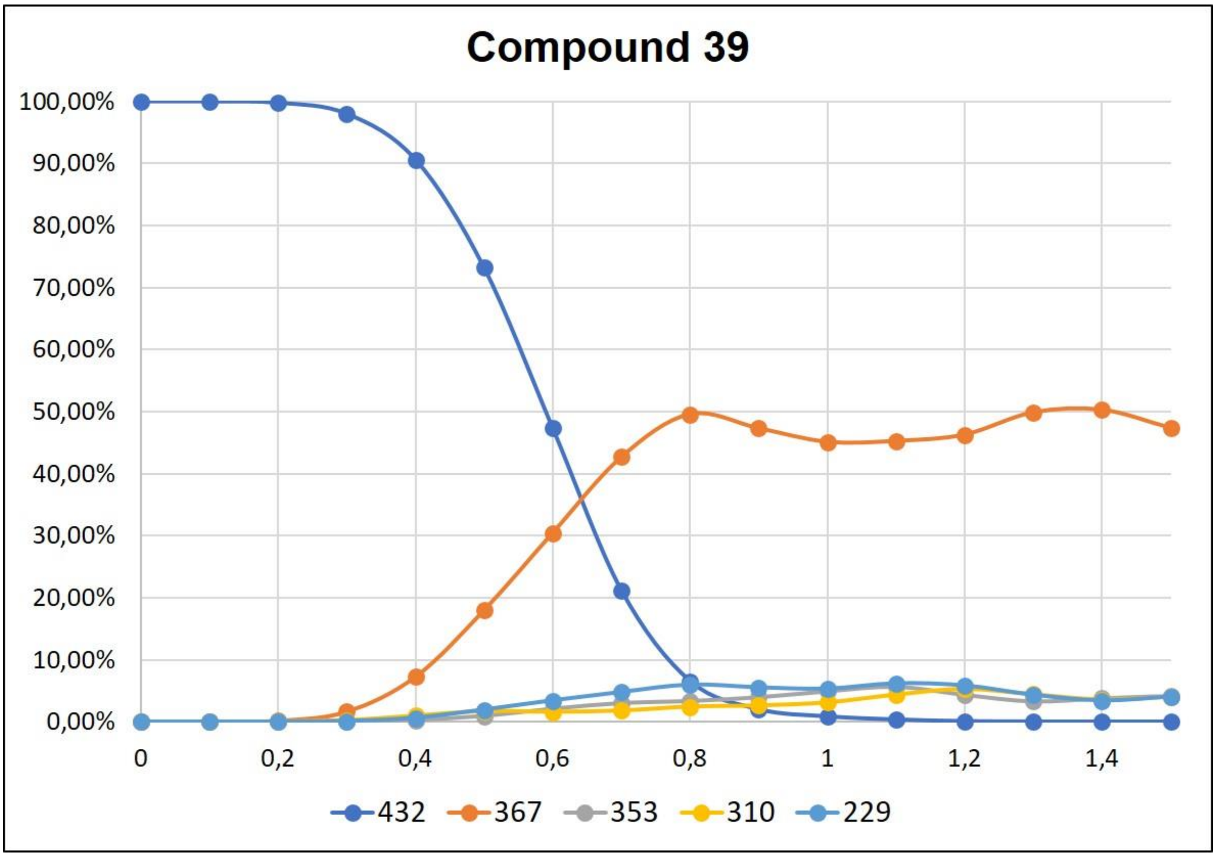

Figure S4. Breakdown curves of compound **39**.

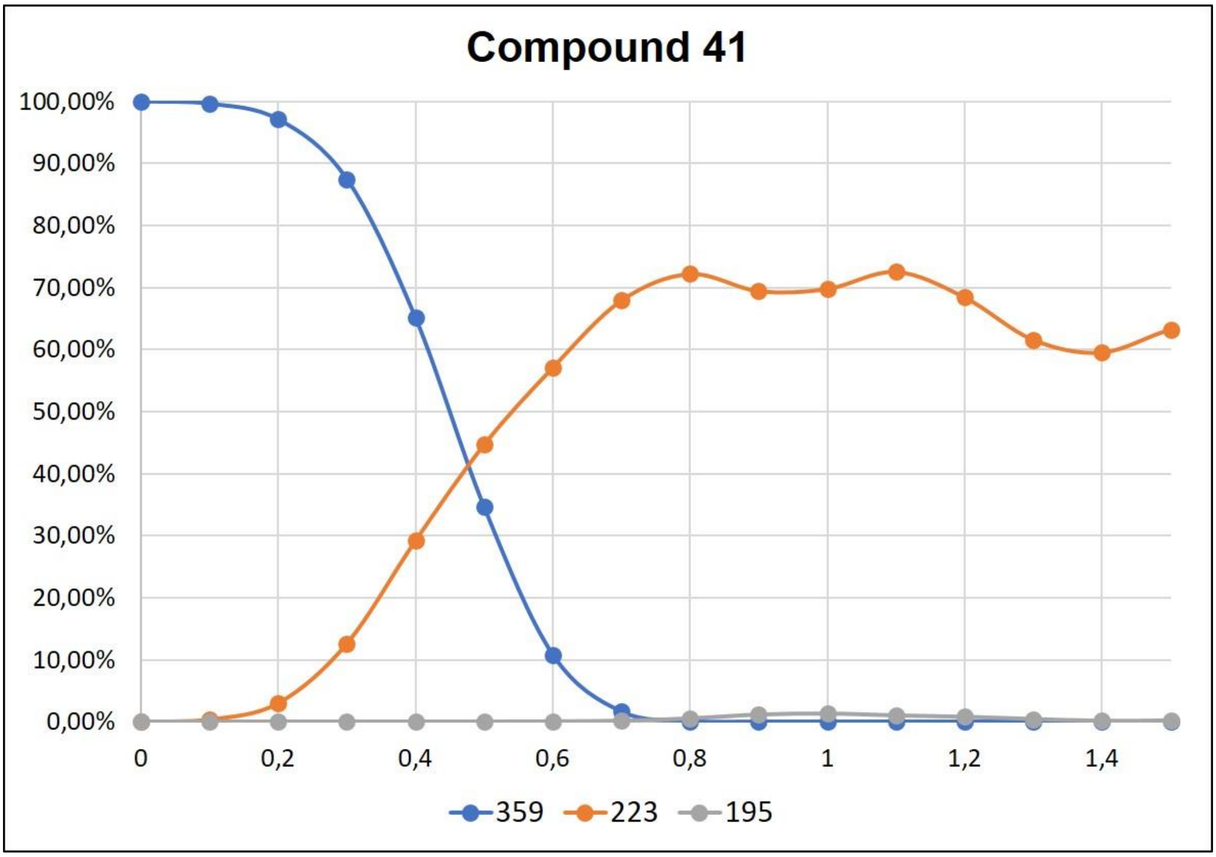

Figure S5. Breakdown curves of compound **41**.

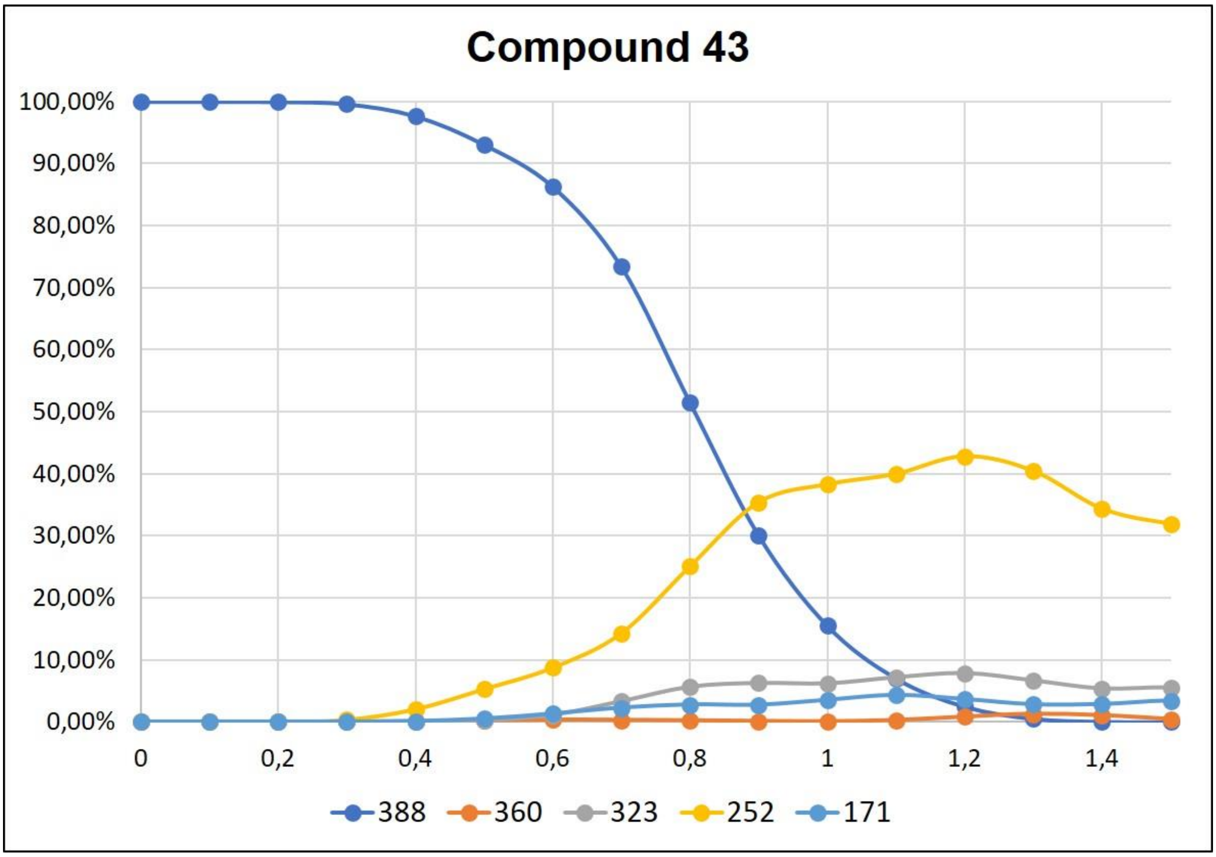

Figure S6. Breakdown curves of compound **43**.
